# Systematic analysis of loss-of-function variants across MODY genes demonstrates gene-specific effects and expands the spectrum of *INS* variants causing MODY

**DOI:** 10.1007/s00125-026-06685-7

**Published:** 2026-03-03

**Authors:** Thomas W. Laver, Aparajita Sriram, Matthew N. Wakeling, Zeynep Şiklar, Farah Kobaisi, Oguzhan Kalyon, Andrew T. Hattersley, Michael N. Weedon, Sarah E. Flanagan, Elisa De Franco, Kevin Colclough, Kashyap A. Patel

**Affiliations:** 1 Department of Clinical and Biomedical Science, University of Exeter, Exeter, UK; 2 Ankara University Faculty of Medicine, Department of Paediatric Endocrinology, Ankara, Turkey; 3 Université Paris Cité, Institut Cochin, CNRS, Inserm, Paris, France; 4 Royal Devon and Exeter NHS Foundation Trust, Exeter, UK

**Keywords:** Gene-burden testing, *INS*, Loss-of-function variants, MODY, MODY gene pathogenicity, Nonsense-mediated decay

## Abstract

**Aims/hypothesis:**

Accurate interpretation of loss-of-function (LOF) variants in MODY genes is essential for diagnosis but remains challenging, particularly for variants that are predicted to escape nonsense-mediated decay (NMD). We aimed to systematically evaluate the pathogenicity of LOF variants, stratified by NMD-triggering and NMD-escape status, across all known MODY genes.

**Methods:**

We analysed ultra-rare LOF variants (minor allele frequency <1 in 10,000) in 5171 individuals of European ancestry with suspected MODY, compared with 155,501 population-based control individuals from UK Biobank. LOF variants in *ABCC8*, *GCK*, *HNF1A*, *HNF4A*, *HNF1B*, *INS*, *KCNJ11*, *NEUROD1*, *PDX1* and *RFX6* were classified as NMD-triggering or NMD-escape. We tested for gene-level enrichment in cases vs controls. For novel associations, we performed replication in additional MODY patients, assessed familial co-segregation, and undertook in silico protein modelling.

**Results:**

LOF variants were significantly enriched in all MODY genes except *ABCC8* and *KCNJ11*. Both NMD-triggering and NMD-escape variants were enriched in *GCK*, *HNF1A* and *HNF4A*, consistent with haploinsufficiency (all *p*<10^−3^). *HNF1B* and *RFX6* showed enrichment only for NMD-triggering variants, while *NEUROD1* and *PDX1* were enriched only for NMD-escape variants. A novel finding was the significant enrichment of only NMD-escape LOF variants in *INS* (OR=181, *p*<10^−5^). Including replication in additional MODY patients, we identified eight families with 17 affected individuals carrying *INS* variants. These variants co-segregated with diabetes (logarithm of the odds score=3), included one de novo case, and were absent from >800,000 population control individuals. Individuals presented with diabetes at a median age of 19 years, had median BMI of 22.9 kg/m^2^, were negative for islet autoantibodies, and had low type 1 diabetes genetic risk scores. Compared with *INS* missense MODY, diagnosis occurred approximately 10 years later in individuals with NMD-escape LOF variants. Protein modelling suggested that *INS* NMD-escape variants produce aberrant proinsulin molecules with unpaired B-chain cysteines, leading to milder misfolding.

**Conclusions/interpretation:**

The pathogenicity of LOF variants in MODY genes depends on gene context and NMD status. Heterozygous NMD-escape LOF variants in *INS* are a novel cause of MODY. These findings provide systematic gene-level evidence to inform variant interpretation guidelines and improve the accuracy of MODY diagnosis in clinical practice.

**Graphical Abstract:**

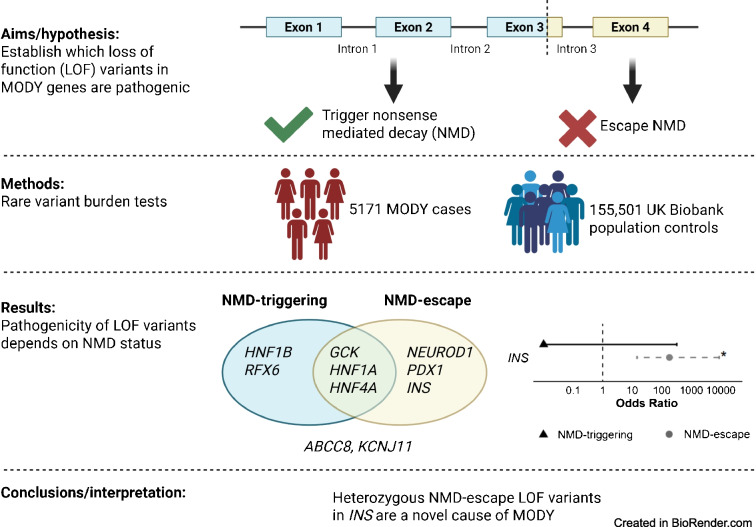

**Supplementary Information:**

The online version contains peer-reviewed but unedited supplementary material available at 10.1007/s00125-026-06685-7.


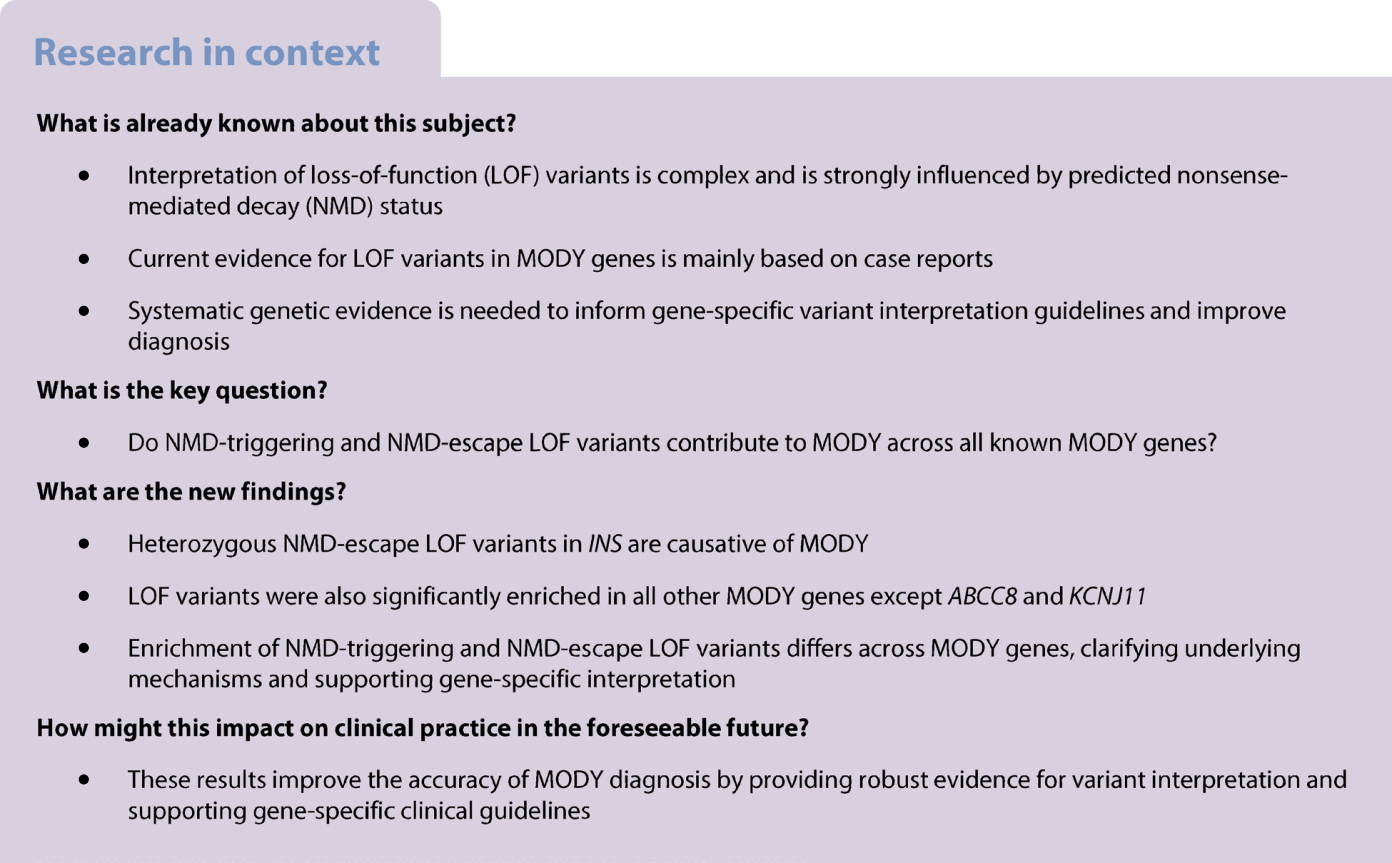


## Introduction

Accurate interpretation of genetic variants in MODY genes is critical for diagnosis but remains a challenge in clinical practice. Variant classification remains challenging due to uncertainty regarding functional impact, the complexity of disease mechanisms, and evolving evidence. This is particularly true for loss-of-function (LOF) variants.

Interpreting LOF variants is complex because their impact on protein function and disease risk depends on their position within the gene. LOF variants typically include nonsense and frameshift variants, canonical splice site changes and exon deletions. LOF variants located more than 50 bp upstream of the final exon–intron junction typically trigger nonsense-mediated decay (NMD), resulting in haploinsufficiency. These are usually pathogenic when haploinsufficiency is a known disease mechanism. However, in the absence of gene-specific evidence, interpretation remains uncertain. In contrast, LOF variants outside the NMD region may avoid mRNA degradation and produce truncated proteins with unpredictable effects. These may retain function, result in complete loss of function, or act in a dominant-negative manner, making classification particularly challenging [1]. Although international guidelines exist for interpreting such variants, they are generic, and there is a strong need for development of gene-specific guidance [2, 3].

Current MODY-specific guidelines for LOF variants are limited to *GCK*, *HNF1A* and *HNF4A*, and further evidence is needed to classify these variants across all MODY genes. There is strong genetic evidence that NMD-triggering LOF variants in some MODY genes (e.g. *GCK*, *HNF1A* and *HNF4A*) cause disease via haploinsufficiency. Evidence for the pathogenicity of NMD-escape LOF variants in MODY is particularly limited. International guidelines recommend classifying NMD-escape LOF variants in *GCK*, *HNF1A* and *HNF4A* as pathogenic [4], but these recommendations are based on case reports rather than robust statistical genetic evidence [5–7]. Isolated case reports also suggest that NMD-escape LOF variants in *PDX1*, *HNF1B*, *RFX6* and *INS* may be disease-causing, but again, systematic evidence is lacking [8–11].

In this study, we performed a burden analysis of rare LOF variants in known MODY genes, comparing individuals with clinically suspected MODY against large control population cohorts. By evaluating both NMD-triggering and NMD-escape LOF variants, we aim to clarify their pathogenicity, contribute to gene-specific interpretation guidelines, and ultimately improve the genetic diagnosis of MODY.

## Methods

### Study populations

#### MODY cohort

We included 5171 unrelated individuals of European ancestry with clinically suspected MODY, who were referred for genetic testing as part of routine clinical care through the Exeter Genomics Laboratory between December 2015 and October 2023. The clinical features of this cohort are summarised in electronic supplementary material (ESM) Table 1. Determination of sex was based on self-reporting. The study was approved by the North Wales Research Ethics Committee (REC reference: 17/WA/0327), and all participants, or their legal guardians, provided written informed consent.

#### UK Biobank

We used the UK Biobank as a population-based control cohort. This prospective UK study recruited individuals aged 40–70 years irrespective of health status, and collected extensive phenotypic and genetic data. For this analysis, we included participants from the 200,000 whole-genome sequencing release (https://biobank.ndph.ox.ac.uk/showcase/label.cgi?id=273, accessed 15 October 2023). All participants gave informed consent, and the UK Biobank Research Ethics Committee approved the use of these data (REC reference: 11/NW/0382).

#### gnomAD

For sensitivity analyses, we used version 3.1.2 of the Genome Aggregation Database (gnomAD; https://gnomad.broadinstitute.org/, accessed 30 September 2023), which comprises whole-genome sequencing data from 76,156 individuals [12]. This version of gnomAD does not include UK Biobank participants.

#### Replication MODY cohort for INS LOF variants and family member testing

To identify additional individuals with candidate *INS* variants, we reviewed two replication groups: (1) individuals of non-European ancestry with suspected MODY who underwent targeted next-generation sequencing (tNGS) from December 2015 and October 2023 (*n*=1692), and (2) all patients from the UK with suspected MODY referred to the Exeter Genomics Laboratory between October 2023 and November 2024 (*n*=505). To evaluate co-segregation, we tested family members for *INS* LOF variants using Sanger sequencing.

### Genetic analysis

#### MODY cohort

We sequenced 2571 individuals using tNGS for all known MODY genes. The remaining 2600 individuals underwent Sanger sequencing for one or more of *GCK* (*n*=941), *HNF1A* (*n*=1292) or *HNF4A* (*n*=602). The tNGS methods have been described in detail previously [13]. Briefly, we used a custom Agilent SureSelect exon-capture panel (Agilent Technologies) targeting known MODY genes. Sequencing was performed using Illumina NextSeq and NovoSeq sequencers. We called variants using the Genome Analysis Toolkit (GATK) [14].

We performed Sanger sequencing using BigDye Terminator version 3.1 (Applied Biosystems). After removing unincorporated dye-terminators, we ran the fragments on an ABI 3730XL capillary DNA sequencer (Applied Biosystems). We called variants using Mutation Surveyor (SoftGenetics) [15], and all included variants were manually checked using the electropherograms. We determined ancestry from tNGS data using an adapted version of the LASER method optimised for targeted panel data [16]. For individuals without tNGS data, we used self-reported ethnicity.

We annotated variants using the following RefSeq transcripts: *ABCC8* (NM_000352.6), *GCK* (NM_000162.5), *HNF1A* (NM_000545.8), *HNF4A* (NM_175914.5), *HNF1B* (NM_000458.4), *INS* (NM_000207.3), *KCNJ11* (NM_000525.3), *NEUROD1* (NM_002500.4), *PDX1* (NM_000209.4) and *RFX6* (NM_173560.4). We performed annotation using Alamut Batch version 1.11 (Interactive Biosoftware) on the GRCh37 genome build and converted coordinates to GRCh38 for comparison with control data.

We calculated logarithm of the odds (LOD) scores to assess co-segregation, following ClinGen guidelines [17]. We used IBDseq (version r1206) to identify haplotypes shared between the individuals with *INS* LOF variants [18].

#### UK Biobank

We analysed whole-genome sequencing data from 155,501 unrelated individuals of European ancestry. We chose genome sequencing over exome sequencing because it provides more uniform coverage across coding regions and reduces technical artefacts [19]. Variant calling was performed on the GRCh38 reference genome, and we annotated variants using Alamut Batch version 1.11. Szustakowski et al [20] have described the sequencing methods in detail; further documentation is available at https://biobank.ctsu.ox.ac.uk/showcase/label.cgi?id=170.

#### gnomAD

We used data from 34,029 non-Finnish European individuals in gnomAD version 3.1.2 as an additional control cohort. The gnomAD team used a BWA–Picard–GATK pipeline [14] for joint variant calling, and applied quality control using the Hail framework [12]. They performed variant calling on GRCh38, and we annotated variants using Alamut Batch version 1.11.

#### Definition of LOF variants

We defined LOF variants as stop-gain, essential splice site or frameshift variants. Based on published criteria [1], we classified LOF variants as NMD-escape if the premature termination codon lay in the final exon or within the last 50 bp of the penultimate exon (ESM Table 2). We classified all other LOF variants as NMD-triggering. For single-exon genes such as *KCNJ11* and *NEUROD1*, variants are generally not expected to be susceptible to NMD, thus all LOF variants in these genes were classified as NMD-escape variants.

### Ultra-rare variant burden analysis

We tested for the gene-level burden of ultra-rare LOF variants with a minor allele frequency (MAF) of <1 in 10,000. We applied strict sample and variant-level quality control to minimise technical artefacts, and included only high-quality variants across all cohorts (see ESM Methods). We restricted the analysis to individuals of European ancestry to reduce population stratification.

We used Fisher’s exact test to compare the frequency of LOF variants between MODY patients and population control individuals, and calculated ORs with 95% CI. To check for test inflation and identify technical artefacts, we included synonymous variants as negative controls. We included *GCK*, *HNF1A* and *HNF4A* as positive controls because there are ClinGen guidelines for these genes for interpretation of NMD-triggering and NMD-escape variants [4].

To maximise power, we combined patients sequenced by Sanger and tNGS when analysing *GCK*, *HNF1A* and *HNF4A*. We first analysed all LOF variants together, then repeated analyses for NMD-triggering and NMD-escape variants separately. A list of LOF variants in MODY genes is given in ESM Table 3 and NMD-escape variants are shown in ESM Fig. 1. We corrected for multiple testing using a Bonferroni-adjusted significance threshold of *p*<0.0025 (0.05/10 genes × 2 variant categories: LOF and synonymous). Our analysis had 80% power to detect ORs of at least 8.9 for variants with a MAF of 1 in 10,000 in *GCK*, *HNF1A* and *HNF4A*, and an OR of 9.8 for the other genes (ESM Table 4).

#### Sensitivity analyses

We performed several sensitivity analyses to test the robustness of our findings. We repeated burden tests using a stricter frequency threshold (MAF <1 in 20,000) and a more lenient frequency threshold (MAF <1 in 5000) to confirm that the results were not influenced by the choice of frequency cut-off. We also repeated the analysis using gnomAD version 3.1.2 as an alternative control cohort to confirm consistency.

### Protein alignment and modelling

We used the Clustal Omega multiple sequence alignment program (EMBL-EBI) to generate alignments for predicted amino acid sequences in *INS* [21]. We used the AlphaFold server (https://alphafoldserver.com/) to generate protein models and visualise them.

## Results

### Both NMD-triggering and NMD-escape LOF variants in *HNF1A*, *HNF4A* and *GCK* are enriched in the MODY cohort

**Fig. 1:**
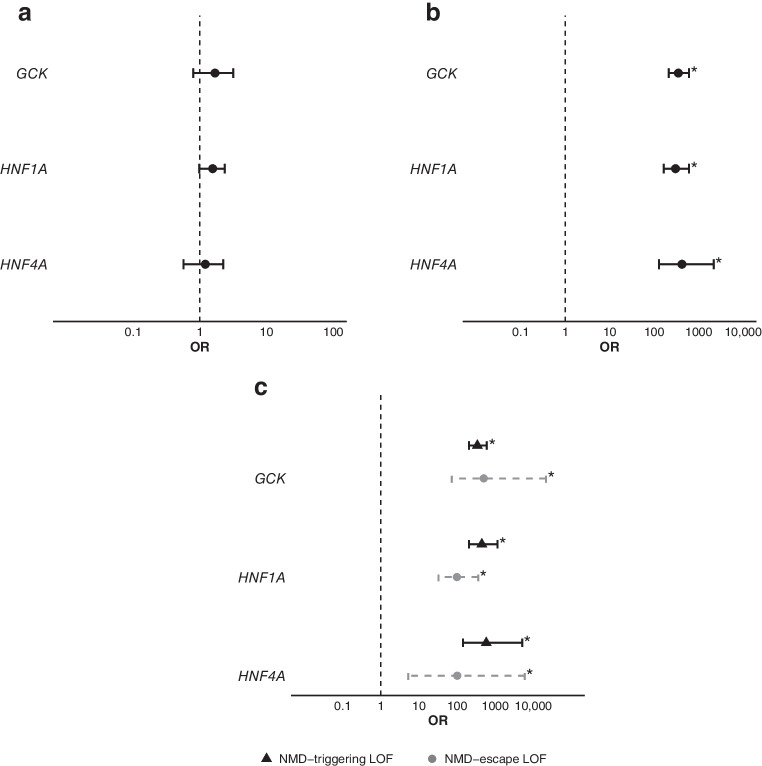
Results of gene-level burden analysis of ultra-rare variants in *GCK*, *HNF1A* and *HNF4A* within the MODY cohort, comparing ultra-rare variants (MAF <1 × 10^−^^4^) between a European-ancestry MODY cohort (*n*=5171) and a UK Biobank control cohort (*n*=155,501). The analyses included: (**a**) synonymous variants, (**b**) LOF variants, and (**c**) LOF variants further divided into those that trigger NMD (black triangles) and those predicted to escape NMD (grey circles). Asterisks indicate statistical significance after correction for multiple testing (*p*<0.0025). ORs and 95% CIs are reported for each association. The allele counts in the MODY cohort and UK Biobank for each gene and LOF category were: NMD-triggering LOF variants: *GCK*, 125 and 17; *HNF1A*, 74 and 7; *HNF4A*, 23 and 2; NMD-escape LOF variants: *GCK*, 11 and 1; *HNF1A*, 12 and 5; *HNF4A*, 2 and 1. The numbers refer to the MODY cohort and the UK Biobank cohort, respectively. Further details are provided in ESM Table 5

The enrichment of rare variants in a disease cohort provides strong genetic evidence of pathogenicity [2]. We therefore assessed the enrichment of ultra-rare LOF variants (stop-gain, essential splice site or frameshift variants; MAF <1 in 10,000) in a MODY cohort compared with 155,501 control individuals. We first focused on the three most common MODY genes, for which robust evidence exists for causality of LOF variants (*GCK*, *HNF1A* and *HNF4A*). We found no enrichment of ultra-rare synonymous variants in these genes, confirming that our analysis was well calibrated (Fig. 1a and ESM Table 5). In contrast, we observed strong enrichment of LOF variants in all three genes in the MODY cohort (all *p*<10^−^^39^; Fig. 1b).

To explore this association further, we analysed LOF variants that are predicted to trigger NMD separately from those predicted to escape NMD. As expected, the NMD-triggering variants (well-established causes of MODY) were strongly enriched in the MODY cohort (ORs=341, 441 and 584 for *GCK*, *HNF1A* and *HNF4A*, respectively; all *p*<10^−^^36^) (Fig. 1c), demonstrating that our analysis was powered to detect true associations.

We also observed marked enrichment of NMD-escape LOF variants in *GCK* (OR=502, *p*<10^−^^19^), *HNF1A* (OR=99, *p*<10^−^^19^) and *HNF4A* (OR=101, *p*<10^−^^3^) (Fig. 1c and ESM Table 5). The effect sizes for NMD-escape and NMD-triggering variants were similar for *GCK* and *HNF4A*, but slightly lower for *HNF1A* (*p*_interaction_=0.046). Taken together, these findings provide strong support for the ClinGen recommendations that LOF variants in *GCK*, *HNF1A* and *HNF4A* should be reported as pathogenic when consistent with the clinical phenotype.

### LOF variants are not enriched in *ABCC8* or *KCNJ11*, while enrichment in other MODY genes depends on NMD status

**Fig. 2:**
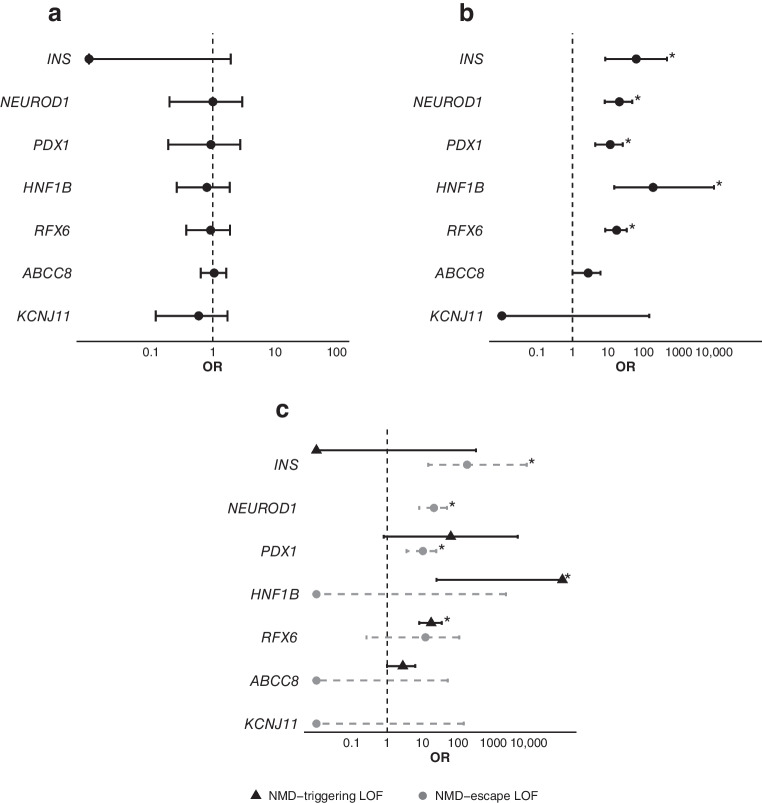
Results of gene-level burden analysis of ultra-rare variants in MODY genes within the MODY cohort, comparing ultra-rare variants (MAF <1 × 10^−^^4^) between a European-ancestry MODY cohort (*n*=2571) and UK Biobank control individuals (*n*=155,501) for the MODY genes *INS*, *NEUROD1*, *PDX1*, *HNF1B*, *RFX6*, *ABCC8* and *KCNJ11*. The analyses included: (**a**) synonymous variants, (**b**) LOF variants, and (**c**) LOF variants further divided into those that trigger NMD (black triangles) and those predicted to escape NMD (grey circles). Asterisks indicate statistical significance after correction for multiple testing (*p*<0.0025). ORs and 95% CIs are reported for each association. The allele counts in the MODY cohort and UK Biobank for each gene and LOF category were: NMD-triggering LOF variants: *ABCC8*, 6 and 131; *HNF1B*, 3 and 0; *INS*, 0 and 2; *PDX1*, 1 and 1; *RFX6*, 11 and 38; NMD-escape LOF variants: *ABCC8*, 0 and 6; *HNF1B*, 0 and 1; *INS*, 3 and 1; *KCNJ11*, 0 and 3; *NEUROD1*, 8 and 23; *PDX1*, 6 and 37; *RFX6*, 1 and 5. The numbers refer to the MODY cohort and the UK Biobank cohort, respectively. Further details are provided in ESM Table 6

We next applied our analysis to the remaining MODY genes, for which the role of LOF variants is less well established and the relevance of NMD-escape vs NMD-triggering variants is unclear. We found no enrichment of ultra-rare synonymous variants in any of these genes, confirming that our analysis was well calibrated (Fig. 2a and ESM Table 6).

We first considered all LOF variants irrespective of NMD status. We found a lack of enrichment in *KCNJ11* (OR=0, *p*>1) and *ABCC8* (OR=2.8, *p*>0.03) (Fig. 2b and ESM Table 6). In contrast, we observed significant enrichment of LOF variants in *HNF1B* (OR=189, *p*<10^−^^4^), *RFX6* (OR=18, *p*<10^−^^10^), *PDX1* (OR=12, *p*<10^−^^5^), *NEUROD1* (OR=21, *p*<10^−7^), and, notably, *INS* (OR=63, *p*<10^−^^4^).

We then split LOF variants by predicted NMD status to assess whether the observed associations differed. As *NEUROD1* and *KCNJ11* are single-exon genes, all LOF variants are expected to escape NMD, so separate analyses were not required. Of the remaining genes, *ABCC8* showed no enrichment for either class (NMD-triggering: OR=2.8, *p*>0.03; NMD-escape: OR=0, *p*=1) (Fig. 2c). By contrast, the other MODY genes displayed distinct patterns, with enrichment driven specifically by NMD-triggering LOF variants in *HNF1B* (OR=∞, *p*<10^−^^5^) and *RFX6* (OR=17, *p*<10^−^^11^); NMD-escape variants were not enriched in either gene (*p*>0.09). Conversely, the enrichment in *PDX1* (OR=10, *p*<10^−^^4^) and *INS* (OR=180, *p*<10^−^^5^) was driven primarily by NMD-escape variants. The *PDX1* results are consistent with previous literature [22], whereas for *INS*, the findings provide the first large-scale evidence that NMD-escape *INS* variants are a novel cause of MODY.

Sensitivity analyses using different frequency thresholds (MAF <1 in 20,000, <1 in 5000 or any frequency) and an alternative control cohort (gnomAD version 3.1.2) produced consistent results (ESM Tables 7–11), supporting the robustness of these findings. Our analyses assess associations at the level of variant groups, not individual variants. Variant-level pathogenicity should be evaluated separately according to American College of Medical Genetics and Genomics and the Association for Molecular Pathology (ACMG/AMP) guidelines [2]. Taken together, these results do not support a causal role for LOF variants in *ABCC8* or *KCNJ11* in MODY. However, they highlight a novel and potential pathogenic role for NMD-escape *INS* variants in MODY.

### Heterozygous NMD-escape LOF variants in *INS* identified in additional MODY patients show strong familial co-segregation and are absent from population databases

Following the novel observation of enrichment of NMD-escape LOF variants in *INS*, we carried out further analyses to assess the strength of genetic evidence for pathogenicity at the variant level.

**Fig. 3:**
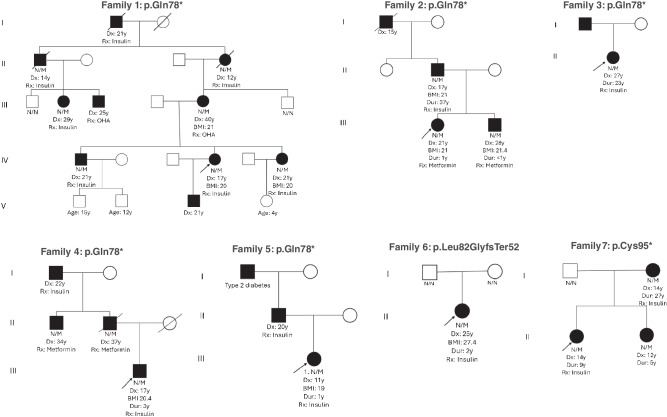
Pedigrees of patients with NMD-escape LOF variants in the *INS* gene within the MODY cohort. Seven families carrying three different variants are shown, with families 1–5 sharing the same p.Gln78* variant. The pedigree for the individual carrying the p.Glu83ValfsTer58 variant is not shown because their family history is unknown. Squares represent male individuals; circles represent female individuals. Black shading indicates individuals diagnosed with diabetes. Arrows indicate the probands. A diagonal strike-through indicates deceased individuals. Labels indicate variant status: N/M indicates individuals who were tested and who were heterozygous for the variant; N/N indicates individuals who were tested and do not carry the variant. BMI is given in kg/m^2^; age is given in years (y). Dur, duration of diabetes; Dx, age at diagnosis; OHA, oral hypoglycaemic agents; Rx, treatment

In our primary cohort of patients of European ancestry referred for MODY genetic testing, we identified three probands carrying the p.Gln78* NMD-escape LOF variant in *INS* (in families 2, 3 and 4 in Fig. 3). To find additional patients, we reviewed all individuals of non-European ancestry with suspected MODY (*n*=1692) and recent referrals to our laboratory for genetic testing. This search identified five additional probands: two with the same p.Gln78* variant and three with other truncating variants: p.Leu82GlyfsTer52, p.Cys95* and p.Glu83ValfsTer58 (in families 1, 5, 6 and 7 in Fig. 3; the pedigree for the individual carrying the p.Glu83ValfsTer58 variant is not shown because their family history is unknown) In total, we identified eight families with NMD-escape *INS* variants, comprising 17 affected individuals (marked N/M in Fig. 3).

None of these variants were present in gnomAD version 4 (*n*=807,162), supporting their rarity in the general population. We confirmed that the p.Leu82GlyfsTer52 variant occurred de novo in the proband (Fig. 3, family 6). We had sufficient genotype data to assess familial co-segregation for the p.Gln78* variant. This variant showed strong co-segregation with diabetes, with a combined LOD score of 3.0 across five families (Fig. 3, families 1–5), consistent with full penetrance in the individuals tested. We assessed the five families with p.Gln78* who had suitable available sequencing data to assess a shared haplotype, but none was detected. Taken together, these findings provide both variant-level and gene-level evidence supporting NMD-escape LOF variants in *INS* as a novel cause of MODY.

### Patients with *INS* NMD-escape LOF variants have a phenotype consistent with a monogenic aetiology

We assessed whether the clinical features of individuals with *INS* NMD-escape protein-truncating variants supported a monogenic cause of diabetes. These individuals had a median age at diagnosis of 19 years, a median HbA_1c_ of 62.8 mmol/mol (7.9%), and a median BMI of 22.9 kg/m^2^ (Table 1). They also lacked islet autoantibodies associated with type 1 diabetes, and their genetic risk for type 1 diabetes was low (median first percentile of the type 1 diabetes population). They also showed evidence of residual insulin secretion, with a median C-peptide of 429.5 pmol/l at a median diabetes duration of 12.9 years. In line with this, only 70.6% were treated with insulin. Together, these features are not consistent with a polygenic aetiology, and support a causal role of *INS* NMD-escape LOF variants for MODY. Interesting, their clinical features were similar to those of the patients with pathogenic *INS* missense variants causing MODY (variants listed in ESM Table 12), except that individuals with *INS* NMD-escape LOF variants were diagnosed 10 years later and had higher random C-peptide, although the difference was not statistically significant (Table 1). These data suggest that these LOF variants are potentially less severe than pathogenic *INS* missense variants.

**Table 1:** Clinical features of MODY patients with NMD-escape LOF variants in *INS* compared to MODY patients with missense variants in *INS*

Characteristic	Patients with *INS* NMD-escape LOF variants (*n*=17)	Patients with *INS* missense variants (*n*=43)	*p* value
Age at diagnosis (years)	19 (17–27)	9 (2–14)	9.3 × 10^−6^
Duration diabetes (years)	12.9 (1.3–20.3)	11.9 (1.9–29.3)	0.5
Female sex	70.6	46.5	0.0004
BMI (kg/m^2^)	22.9 (20.4–27.0)	22.2 (19.8–26.2)	0.6
Parent with diabetes	93.8	71.9	0.1
HbA1c (mmol/mol)	62.8 (47.5–91.3)	62.8 (53.0–107.7)	0.9
HbA_1c_ (%)	7.9 (6.5–10.5)	7.9 (7.0–12.0)	0.9
C-peptide (pmol/l)	429.5 (150.0–1119.3)	300 (115.3–554.8)	0.4
Islet autoantibody-positive	0	5	1
T1DGRS (centile)	1.2 (0.7–16.8)	5 (1.5–15.7)	0.05
Current insulin treatment	70.6	90.2	0.1

Data are medians (IQR) for continuous variables and % for categorical variables

A list of missense variants is provided in ESM Table 12

T1DGRS refers to Type 1 Diabetes Genetic Risk score [38]

### MODY-associated LOF *INS* variants potentially lead to milder misfolding

To investigate the mechanisms by which NMD-escape LOF variants in *INS* cause MODY, we performed sequence alignment and structural modelling of our newly identified variants together with all published LOF variants.

**Fig. 4:**
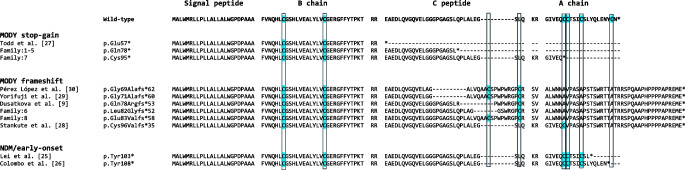
Predicted protein sequences of *INS* NMD-escape LOF variants. The wild-type *INS* sequence is shown at the top for reference. MODY-associated stop-gain and frameshift NMD-escape LOF variants identified in our cohort or reported in the literature are included. Published NMD-escape LOF variants linked to neonatal diabetes (NDM) or early-onset diabetes are also shown. Conserved cysteine residues are highlighted in blue; asterisks indicate a stop codon

Correct folding of insulin requires precise disulfide bonding between cysteines in the B and A chains. All stop-gain MODY variants truncate the entire A chain, leaving the B-chain cysteines unpaired and incapable of forming disulfide bonds (Fig. 4 and ESM Fig. 2). Similarly, MODY frameshift variants uniformly alter the reading frame by +2, abolishing the native A chain and extending translation into the 3′ UTR where all variants terminate. Although these frameshifts introduce one or two cysteines, structural modelling indicates that the new residues are potentially positioned too far from the B-chain cysteines to form aberrant disulfide bonds (range 6.9–20.4 Å; the typical distance for wild-type *INS* is approximately 4.8 Å) (ESM Fig. 2) [23, 24].

These data indicate that LOF MODY variants consistently result in unpaired B-chain cysteines. This may cause relatively modest misfolding that is nonetheless sufficient to activate the unfolded protein response and result in beta cell death leading to young-onset diabetes, although less severe in both effect and outcome compared with variants causing neonatal diabetes. Consistent with this interpretation, no heterozygous frameshift variants have been reported in neonatal diabetes. By contrast, only two heterozygous truncating variants have been described, and both were NMD-escape variants (p.Tyr103* and p.Tyr108*) [25, 26]. These variants truncate late in the A chain, preserving the first three cysteines but removing the fourth. This configuration is predicted to permit aberrant crosslinking of B-chain cysteines, leading to severe misfolding, a stronger unfolded protein response and severe beta cell death, leading to neonatal diabetes (ESM Fig. 2).

## Discussion

In this study, we provide gene-level evidence for the pathogenicity of both NMD-triggering and NMD-escape LOF variants across all known MODY genes. We show that the pathogenic impact of a LOF variant depends on whether it triggers NMD, and that this varies by gene. Specifically, we demonstrate that NMD-escape LOF variants in *INS* are a novel cause of MODY.

Our study expands the spectrum of *INS* variants known to cause MODY. Our conclusion is supported by strong gene- and variant-level data. We observed significant enrichment in MODY patients compared with population control individuals (OR=181, *p*<10^−5^), clear co-segregation in families (LOD score=3.0), and one de novo occurrence. In total, we identified four variants in eight families, none of which are present in a population database comprising 807,162 individuals (gnomAD version 4, which includes the UK Biobank samples). The clinical features of affected individuals were consistent with monogenic diabetes, and resembled those seen in patients with pathogenic MODY-causing *INS* missense variants, although diagnosis occurred approximately 10 years later. Six NMD-escape *INS* variants have been described previously in diabetes patients (ESM Table 13 and ESM Fig. 3). Three were reported as part of larger cohorts without detailed phenotyping [27–29], while three were described in individual case reports [9, 30, 31]. The patients were diagnosed at between 7 and 36 years of age (mean 20 years), consistent with our cohort. Together, this evidence establishes heterozygous *INS* NMD-escape LOF variants as a cause of MODY that should be reported in diagnostic testing. However, the lack of enrichment of NMD-triggering variants in our study and lack of diabetes in parents of infants with recessive neonatal diabetes due to NMD-triggering variants suggest that variants that cause haploinsufficiency do not cause MODY and should not be reported [32].

We hypothesise that NMD-escape LOF variants in *INS* act by generating aberrant proinsulin molecules with unpaired B-chain cysteines. Variants with an intact A chain but loss of at least one cysteine show abnormal disulfide bond formation, producing severe misfolding and neonatal diabetes. In contrast, variants that remove or disrupt the entire A chain leave unpaired B-chain cysteines, leading to milder misfolding (ESM Fig. 2). Our findings are consistent with previous in vitro work showing that truncated insulin containing only the B chain fails to interact with wild-type insulin. Together, these data support a model in which MODY-associated variants are dominant-negative and cause milder misfolding without toxic gain of function, resulting in a weaker unfolded protein response and slower beta cell loss. This mechanism remains a hypothesis that requires confirmation in cellular and in vivo systems.

Our results also clarify the role of LOF variants in *ABCC8* and *KCNJ11*. We found no evidence of enrichment, questioning the role of heterozygous LOF variants in these genes in MODY. This finding requires further follow-up variant-level genetic evidence to conclusively refute a causal role of these variants in MODY. However, our findings are consistent with widely reported disease mechanisms of these genes, whereby activating variants in *ABCC8* and *KCNJ11* cause neonatal diabetes and MODY, while recessive LOF variants cause congenital hyperinsulinism [33, 34]. A recent study has suggested that heterozygous LOF *ABCC8* variants could play a role in MODY [35]. However, our results do not support haploinsufficiency as the underlying mechanism for MODY.

We identified additional gene-specific patterns. NMD-escape LOF variants in *PDX1* were associated with MODY. We observed three frameshift variants disrupting the homeodomain, which is encoded by the second and last exon, and three frameshift/stop-gain variants occurring after the homeodomain; none of these patients carried other known pathogenic variants. These findings are consistent with the dominant-negative mechanism previously described [36]. Our study was underpowered to assess NMD-triggering protein-truncating variants in isolation, as only one such case was present in the MODY cohort (p.Cys18*, female, diagnosed in their teens, without a known alternative genetic diagnosis), with an OR of 63 (0.8, 4941; *p*=0.03). These results suggest that further variant-level evidence and larger studies are needed to clarify the role of NMD-triggering variants in *PDX1*. For *HNF1B* and *RFX6*, enrichment was confined to NMD-triggering LOF variants, with no evidence supporting pathogenicity of NMD-escape variants. Although isolated *RFX6* NMD-escape variants have been reported [37], our results suggest these findings should be interpreted cautiously. In contrast, we found enrichment of both NMD-triggering and NMD-escape protein-truncating variants in *GCK*, *HNF1A* and *HNF4A*, consistent with ClinGen recommendations for reporting such variants. The presence of functional domains in the NMD-escape regions of *GCK* and *HNF1A* supports the functional importance of this region of those genes, although *HNF4A* lacks a defined functional domain in the NMD-escape region, and the basis of pathogenicity remains to be determined. Overall, our results provide mechanistic insights into MODY gene action.

Our study has limitations. Although we leveraged one of the largest available MODY cohorts, we had power only to detect associations with ORs ≥9.8 at an allele frequency of 0.0001. The number of variants in some genes, particularly within the NMD-escape regions, was small, which restricted our ability to detect weaker associations and reduced the precision of our estimates. Given the relatively short span of NMD-escape regions, future studies with even larger sample size will be essential to more accurately quantify the impact of these variants on diabetes risk. Also, we cannot exclude the possibility that some of the genes harbour ultra-rare or low-penetrance LOF variants that were undetected in our analysis. Our burden testing was limited to individuals of European ancestry, in whom statistical power was greatest. Future work should assess these associations across diverse ancestries. We do not consider sex to be a variable that will have affected our analysis, and thus our results are applicable to all sexes; however, this possibility was not explicitly analysed. We used publicly available population control individuals that are not filtered by diabetes status and therefore include individuals with diabetes. This approach reflects standard methodology in rare-variant enrichment analyses, in which population control individuals approximate the general at-risk population rather than a strictly disease-free subset. Including all individuals reduces the likelihood of false-positive signals driven by very low-penetrance variants. Synonymous variant analyses supported the robustness of our variant quality control pipeline.

In conclusion, we provide strong genetic evidence that heterozygous NMD-escape LOF variants in *INS* are a novel cause of MODY. Our systematic analysis across all known MODY genes provides robust statistical evidence to guide gene-specific variant interpretation, support ClinGen recommendations, and improve diagnosis of monogenic diabetes.

## Supplementary Information

Below is the link to the electronic supplementary material.

ESM (PDF 2039 KB)

## Data Availability

Data on the MODY cohort are available through collaboration to experienced teams working on approved studies examining the mechanisms, diagnosis and treatment of diabetes and other beta cell disorders. Requests for collaboration will be considered by a steering committee following an application to the Genetic Beta Cell Research Bank (https://www.diabetesgenes.org/current-research/genetic-beta-cell-research-bank/). Contact by email should be directed to the corresponding author. Data from the UK Biobank are available to approved researchers (https://www.ukbiobank.ac.uk/use-our-data/apply-for-access/). GnomAD data are publicly available (https://gnomad.broadinstitute.org/).

## Abbreviations

gnomAD: Genome Aggregation Database
LOD: Logarithm of the odds
LOF: Loss of function
MAF: Minor allele frequency
NMD: Nonsense-mediated decay
tNGS: Targeted next-generation sequencing
