# Supplementary material for: Systematic analysis of loss-of-function variants across MODY genes demonstrates gene-specific effects and expands the spectrum of INS variants causing MODY
Source: Diabetologia. 2026 Mar 3;69(6):1569–80. doi: 10.1007/s00125-026-06685-7 (PMC13109276; doi:10.1007/s00125-026-06685-7)
Supplement: Supplementary file 1 — ESM (PDF 2039 KB) [file 125_2026_6685_MOESM1_ESM.pdf]

ESM Methods

Quality control

MODY cohort

For our tNGS sequenced samples, we performed a rigorous quality control (QC) process at the sample, variant, and genotype levels to ensure the accuracy and consistency of sequencing data obtained from different platforms. Recognizing the potential for discrepancies between sequencing technologies, we applied stringent filters to minimize technical artifacts and enhance reliability. We began by excluding six samples that had a genotype missingness rate greater than 2%.

We then applied multiple filters at the variant level. We removed variants with a strand bias FS score greater than 60, a quality-by-depth (QD) score less than 2, a ReadPosRankSum score below -8, or a MQRankSum score below -12.5. We also excluded variants with a mapping quality less than 40, a read depth below 20, or a genotype quality below 20. To account for allelic imbalance, we removed variants for which a binomial test yielded a p-value less than 0.001. We additionally excluded variants located in the *HNF1A* c-insertion region (chr12:120994310-120994335), which is known to produce false positives. Finally, we removed any genotypes with a missingness rate greater than 2%.

After applying all quality control steps, we retained 554 high-quality variants for downstream analyses.

UK Biobank

We applied the same quality control strategy to the UK Biobank dataset, with minor adjustments to reflect its use of whole-genome sequencing rather than targeted gene panels. Specifically, we lowered the read depth threshold to 15 for variant exclusion. We also removed variants with an AAscore below 0.5, a metric generated by Graphtyper that estimates the likelihood of a variant being a true positive. (1) Furthermore, we excluded any variants located in low-complexity regions as flagged by gnomAD. To maintain consistency between cohorts, we excluded any variant that failed quality control in either the MODY cohort or the UK Biobank from both datasets.

gnomAD

We used publicly available gnomAD data, which had already undergone sample and variant-level quality control. In addition to these existing filters, we excluded variants that were located in regions with low coverage, defined as ≤10× in more than 20% of samples. We also removed variants that were filtered by gnomAD or flagged as being located in low-complexity regions. As with the MODY and UK Biobank datasets, we removed any variant that failed quality control in either the MODY cohort or gnomAD from both datasets prior to analysis.

Reference:

1. Halldorsson B V., Eggertsson HP, Moore KHS, Hauswedell H, Eiriksson O, Ulfarsson MO, et al. The sequences of 150,119 genomes in the UK Biobank. Nature. 2022;607(7920).

ESM Results

ESM Table 1: Characteristics of MODY cohort

CHARACTERISTICS	MODY COHORT
N	5,171
Age of diagnosis of diabetes, years	22 (15-20)
Female Sex, %	59.2
Age at recruitment, years	29.6 (18.9-38.6)
BMI, kg/m^2	25.2 (22.1-29.6)
Parents with diabetes, %	69.4
HbA1c, %	7.3 (6.4-9.3)
On Insulin treatment, %	45.7

Median (IQR) for continuous variable and n (%) for categorical data

ESM Table 2: NMD-escape regions in each MODY gene from Ensembl (GRCh38)

Gene	Transcript	No. of exons	Direction	Chr	Last exon		Last 50 bp of penultimate exon	
					start	end	start	end
INS	NM_000207.3	2 (+1 noncoding)	3'>5'	11	2159997	2159852	2160835	2160785
PDX1	NM_000209.4	2	5'>3'	13	27924256	27924701	27920494	27920544
HNF1A	NM_000545.8	10	5'>3'	12	121001065	121001192	120999577	120999627
HNF4A	NM_175914.5	10	5'>3'	20	44429524	44429665	44428437	44428487
GCK	NM_000162.5	10	3'>5'	7	44145280	44145136	44145547	44145497
RFX6	NM_173560.4	19	5'>3'	6	116931331	116931506	116928921	116928971
ABCC8	NM_000352.6	39	3'>5'	11	17393128	17392991	17393747	17393697
HNF1B	NM_000458.4	9	3’>5’	17	37687392	37687372	37699126	37699076

ESM Table 3: List of LOF function variants in MODY genes seen in the MODY cohort (n=5,171)

Gene	Variant	cNomen	pNomen	Allele count in MODY cohort
ABCC8	11:17393125:G:A	NM_001287174.1:c.4615C>T	p.Arg1539*	0
ABCC8	11:17393129:C:A	NM_001287174.1:c.4612-1G>T	p.?	0
ABCC8	11:17394358:G:A	NM_001287174.1:c.4456C>T	p.Gln1486*	0
ABCC8	11:17395277:T:C	NM_001287174.1:c.4311-2A>G	p.?	1
ABCC8	11:17395611:G:A	NM_001287174.1:c.4309C>T	p.Arg1437*	0
ABCC8	11:17395626:AGAGGACGG:A	NM_001287174.1:c.4286_4293del	p.Pro1429Leufs*8	0
ABCC8	11:17395667:AG:A	NM_001287174.1:c.4252del	p.Leu1418Cysfs*43	0
ABCC8	11:17395855:C:A	NM_001287174.1:c.4198G>T	p.Glu1400*	0
ABCC8	11:17395911:GT:G	NM_001287174.1:c.4141del	p.Thr1381Profs*80	0
ABCC8	11:17396914:AC:A	NM_001287174.1:c.4122+1del	p.?	1
ABCC8	11:17396915:C:T	NM_001287174.1:c.4122+1G>A	p.?	0
ABCC8	11:17397191:A:G	NM_001287174.1:c.3991+2T>C	p.?	0
ABCC8	11:17398337:G:A	NM_001287174.1:c.3756+2C>T	p.?	0
ABCC8	11:17398344:G:A	NM_001287174.1:c.3751C>T	p.Arg1251*	0
ABCC8	11:17398410:CG:C	NM_001287174.1:c.3684del	p.Glu1229Asnfs*37	0
ABCC8	11:17398440:AC:A	NM_001287174.1:c.3654del	p.Arg1218Serfs*48	0
ABCC8	11:17398442:C:G	NM_001287174.1:c.3654-1G>C	p.?	0
ABCC8	11:17404511:C:T	NM_001287174.1:c.3560+1G>A	p.?	0
ABCC8	11:17404559:CA:C	NM_001287174.1:c.3512del	p.Leu1171Argfs*38	3
ABCC8	11:17404623:CAG:C	NM_001287174.1:c.3447_3448del	p.Val1151Leufs*43	0
ABCC8	11:17404642:TC:T	NM_001287174.1:c.3429del	p.Ser1144Alafs*65	0
ABCC8	11:17406690:C:T	NM_001287174.1:c.3264G>A	p.Trp1088*	0
ABCC8	11:17406900:GCAGTTCCTGGCTGCAGGGGT:G	NM_001287174.1:c.3133_3152del	p.Thr1045Leufs*63	1
ABCC8	11:17406943:C:T	NM_001287174.1:c.3110G>A	p.Trp1037*	0
ABCC8	11:17407058:G:A	NM_001287174.1:c.2995C>T	p.Arg999*	0
ABCC8	11:17407059:C:T	NM_001287174.1:c.2994G>A	p.Trp998*	0
ABCC8	11:17407353:C:T	NM_001287174.1:c.2923+1G>A	p.?	0
ABCC8	11:17407454:C:T	NM_001287174.1:c.2824-1G>A	p.?	0
ABCC8	11:17408391:C:T	NM_001287174.1:c.2823+1G>A	p.?	0
ABCC8	11:17408415:G:A	NM_001287174.1:c.2800C>T	p.Arg934*	0
ABCC8	11:17410621:CAGAT:C	NM_001287174.1:c.2588_2591del	p.His863Argfs*5	0
ABCC8	11:17412716:G:A	NM_001287174.1:c.2509C>T	p.Arg837*	0
ABCC8	11:17412747:C:G	NM_001287174.1:c.2479-1G>C	p.?	0
ABCC8	11:17413479:C:T	NM_001287174.1:c.2394-1G>A	p.?	0
ABCC8	11:17416966:C:A	NM_001287174.1:c.2223-1G>T	p.?	0
ABCC8	11:17416966:C:T	NM_001287174.1:c.2223-1G>A	p.?	0
ABCC8	11:17427155:C:T	NM_001287174.1:c.2117-1G>A	p.?	0
ABCC8	11:17427865:A:G	NM_001287174.1:c.2116+2T>C	p.?	0
ABCC8	11:17427870:G:A	NM_001287174.1:c.2113C>T	p.Arg705*	0
ABCC8	11:17428407:T:A	NM_001287174.1:c.1924-2A>T	p.?	0
ABCC8	11:17428594:G:A	NM_001287174.1:c.1894C>T	p.Gln632*	0
ABCC8	11:17428608:TG:T	NM_001287174.1:c.1879del	p.His627Metfs*20	0
ABCC8	11:17430839:G:A	NM_001287174.1:c.1792C>T	p.Arg598*	0
ABCC8	11:17442719:C:A	NM_001287174.1:c.1630+1G>T	p.?	1

ABCC8	11:17442719:C:T	NM_001287174.1:c.1630+1G>A	p.?	0
ABCC8	11:17442883:C:T	NM_001287174.1:c.1468-1G>A	p.?	0
ABCC8	11:17443306:C:CAA	NM_007174.1:c.1337_1338dup	p.Val447Leufs*4	0
ABCC8	11:17448558:C:T	NM_001287174.1:c.1290G>A	p.Trp430*	0
ABCC8	11:17448563:T:TGAGCTGATTGGTGTCGATGGCAACCAGATTA	NM_001287174.1:c.1254_1284dup	p.Met429*	0
ABCC8	11:17453117:A:G	NM_001287174.1:c.1176+2T>C	p.?	0
ABCC8	11:17461616:G:T	NM_001287174.1:c.789C>A	p.Tyr263*	0
ABCC8	11:17461663:G:A	NM_001287174.1:c.742C>T	p.Arg248*	0
ABCC8	11:17461679:CT:C	NM_001287174.1:c.725del	p.Lys242Argfs*16	0
ABCC8	11:17461709:C:T	NM_001287174.1:c.696G>A	p.Trp232*	0
ABCC8	11:17461750:G:A	NM_001287174.1:c.655C>T	p.Gln219*	0
ABCC8	11:17461826:C:G	NM_001287174.1:c.580-1G>C	p.?	0
ABCC8	11:17463589:C:T	NM_001287174.1:c.428G>A	p.Trp143*	0
GCK	7:44145165:AGGCCACCGCCGAGACCAGGGCCGCGCCCC:A	NM_000162.5:c.1340_1368del	p.Arg447LeufsTer2	1
GCK	7:44145173:GC:G	NM_000162.5:c.1360del	p.Ala454Argfs*160	2
GCK	7:44145190:GC:G	NM_000162.5:c.1343del	p.Gly448AlafsTer166	1
GCK	7:44145210:C:CCGACT	NM_000162.5:c.1319_1323dup	p.Glu442SerfsTer174	1
GCK	7:44145210:CCGACT:C	NM_000162.5:c.1319_1323del	p.Glu440GlyfsTer17	1
GCK	7:44145216:C:A	NM_000162.5:c.1318G>T	p.Glu440Ter	1
GCK	7:44145498:TG:T	NM_000162.5:c.1251del	p.Ser418AlafsTer13	1
GCK	7:44145505:CAGCTTGTACACGG:C	NM_000162.5:c.1232_1244del	p.Ser411CysfsTer16	1
GCK	7:44145510:T:A	NM_000162.5:c.1240A>T	p.Lys414Ter	1
GCK	7:44145523:AT:A	NM_000162.5:c.1226del	p.Asp409ValfsTer22	1
GCK	7:44145551:TC:T	NM_000162.5:c.1198del	p.Asp400ThrfsTer2	1
GCK	7:44145612:G:GCGCAGCGCGCGTAGACACGCTCT	NM_000162.5:c.1115_1137dup	p.His380ArgfsTer30	1
GCK	7:44145629:A:ACG	NM_000162.5:c.1119_1120dup	p.Val374AlafsTer29	1
GCK	7:44145654:T:TGTCGCTC	NM_000162.5:c.1095_1096insGAGCGAC	p.Ile366GlufsTer95	1
GCK	7:44145659:C:CAGTTA	NM_000162.5:c.1090_1091insTAACT	p.Cys364LeufsTer40	1
GCK	7:44145671:G:T	NM_000162.5:c.1079C>A	p.Ser360Ter	3
GCK	7:44146462:C:T	NM_000162.5:c.1019+1G>A	p.?	1
GCK	7:44146463:CTCTCCACCTGCGACACGAA:C	NM_000162.5:c.1000_1018del	p.Phe334AlafsTer13	1
GCK	7:44146475:G:T	NM_000162.5:c.1007C>A	p.Ser336Ter	1
GCK	7:44146515:G:A	NM_000162.5:c.967C>T	p.Gln323Ter	1
GCK	7:44146518:C:A	NM_000162.5:c.964G>T	p.Glu322*	0
GCK	7:44146528:CCCGTGGAAGAGCAGGTTTTCGTCCACGAG:C	NM_000162.5:c.925_953del	p.Leu309GlyfsTer140	1
GCK	7:44146543:GT:G	NM_000162.5:c.938del	p.Asn313ThrfsTer40	1
GCK	7:44146566:G:GCACA	NM_000162.5:c.912_915dup	p.Leu306CysfsTer154	1
GCK	7:44146585:GC:G	NM_000162.5:c.896del	p.Gly299AlafsTer54	2
GCK	7:44146619:C:T	NM_000162.5:c.864-1G>A	p.?	9
GCK	7:44147657:G:A	NM_000162.5:c.856C>T	p.Gln286Ter	1
GCK	7:44147658:ACCGGGGTTTGCAGAGCTCTCGTCCAC:A	NM_000162.5:c.829_854del	p.Val277Serfs*5	1
GCK	7:44147660:CG:C	NM_000162.5:c.852del	p.Gly285ValfsTer9	1
GCK	7:44147678:C:A	NM_000162.5:c.835G>T	p.Glu279Ter	1

GCK	7:44147680:TC:T	NM_000162.5:c.832del	p.Asp278ThrfsTer16	1
GCK	7:44147684:C:CCAGG	NM_000162.5:c.825_828dup	p.Val277Profs*15	1
GCK	7:44147728:T:TCC	NM_000162.5:c.784del	p.Asp262ThrfsTer32	1
GCK	7:44147728:TC:T	NM_000162.5:c.783_784dup	p.Asp262GlyfsTer33	1
GCK	7:44147733:GA:G	NM_000162.5:c.779del	p.Phe260SerfsTer34	1
GCK	7:44147755:AC:A	NM_000162.5:c.757del	p.Val253SerfsTer41	1
GCK	7:44147773:T:GC	NM_000162.5:c.739del	p.Asp247ThrfsTer47	1
GCK	7:44147773:TC:T	NM_000162.5:c.740delinsGC	p.Asp247GlyfsTer28	1
GCK	7:44149759:C:T	NM_000162.5:c.679+1G>A	p.?	3
GCK	7:44149779:G:T	NM_000162.5:c.660C>A	p.Cys220Ter	2
GCK	7:44149784:G:A	NM_000162.5:c.655C>T	p.Gln219*	4
GCK	7:44149794:G:C	NM_000162.5:c.645C>G	p.Tyr215*	4
GCK	7:44149794:G:T	NM_000162.5:c.645C>A	p.Tyr215Ter	5
GCK	7:44149858:TC:T	NM_000162.5:c.580del	p.Asp194ThrfsTer10	1
GCK	7:44149859:CCTGCCAAGAAGCA:C	NM_000162.5:c.580-13_580-1del	p.?	0
GCK	7:44149992:G:A	NM_000162.5:c.556C>T	p.Arg186*	23
GCK	7:44150048:C:T	NM_000162.5:c.500G>A	p.Trp167Ter	1
GCK	7:44150939:CCCTCCACCCGGCCCA:C	NM_000162.5:c.483+2_483+16del	p.?	14
GCK	7:44150954:A:G	NM_000162.5:c.483+2T>C	p.?	1
GCK	7:44151002:A:AGG	NM_000162.5:c.435_436dup	p.Leu146Profs*10	3
GCK	7:44152268:CA:C	NM_000162.5:c.363+2del	p.?	0
GCK	7:44152275:TCAGCAGTG:T	NM_000162.5:c.351_358del	p.Thr118Aspfs*8	1
GCK	7:44152318:G:A	NM_000162.5:c.316C>T	p.Gln106*	3
GCK	7:44152320:T:TGTTTGGTCTCC	NM_000162.5:c.313_314insGGAGACCAAAC	p.His105ArgfsTer15	1
GCK	7:44152332:AC:A	NM_000162.5:c.301del	p.Val101Ter	1
GCK	7:44152337:C:T	NM_000162.5:c.297G>A	p.Trp99*	2
GCK	7:44152342:GC:AT	NM_000162.5:c.291_292delinsAT	p.Gln98Ter	1
GCK	7:44152416:TC:T	NM_000162.5:c.217del	p.Asp73Thrfs*14	1
GCK	7:44152426:C:T	NM_000162.5:c.209-1G>A	p.?	0
GCK	7:44153299:A:G	NM_000162.5:c.208+2T>C	p.?	0
GCK	7:44153326:G:T	NM_000162.5:c.183C>A	p.Tyr61*	6
GCK	7:44153377:GC:G	NM_000162.5:c.131del	p.Gly44AlafsTer2	2
GCK	7:44153430:C:A	NM_000162.5:c.79G>T	p.Glu27*	3
GCK	7:44153433:G:A	NM_000162.5:c.76C>T	p.Gln26Ter	1
GCK	7:44153464:C:T	NM_000162.5:c.46-1G>A	p.?	1
GCK	7:44188908:C:A	NM_000162.5:c.45+1G>T	p.?	3
HNF1A	12:120978893:G:GC	NM_000545.6:c.130dup	p.Leu44Profs*16	1
HNF1A	12:120978928:C:T	NM_000545.6:c.160C>T	p.Arg54*	2
HNF1A	12:120978951:CA:C	NM_000545.6:c.185del	p.Asn62Metfs*93	0
HNF1A	12:120979064:AC:A	NM_000545.6:c.298del	p.Gln100Argfs*55	1
HNF1A	12:120979093:C:T	NM_000545.6:c.325C>T	p.Gln109*	1
HNF1A	12:120988872:C:G	NM_000545.6:c.366C>G	p.Tyr122*	1
HNF1A	12:120988909:GA:G	NM_000545.6:c.404del	p.Asp135Valfs*20	3
HNF1A	12:120988916:CTG:C	NM_000545.8:c.411_412del	p.Gly138ProfsTer49	1

HNF1A	12:120988939:T:TC	NM_000545.8:c.436dup	p.Gln146ProfsTer42	1
HNF1A	12:120988978:A:T	NM_000545.8:c.472A>T	p.Lys158Ter	1
HNF1A	12:120988984:GC:G	NM_000545.6:c.480del	p.Ala161Profs*25	1
HNF1A	12:120989000:G:A	NM_000545.8:c.494G>A	p.Trp165Ter	1
HNF1A	12:120989017:C:T	NM_000545.8:c.511C>T	p.Arg171Ter	1
HNF1A	12:120989032:C:T	NM_000545.6:c.526C>T	p.Gln176*	2
HNF1A	12:120989033:G:A	NM_000545.8:c.526+1G>A	p.?	3
HNF1A	12:120989033:G:C	NM_000545.6:c.526+1G>C	p.?	1
HNF1A	12:120989033:G:GT	NM_000545.8:c.526+2dup	p.?	1
HNF1A	12:120993518:A:G	NM_000545.6:c.527-2A>G	p.?	1
HNF1A	12:120993519:G:A	NM_000545.6:c.527-1G>A	p.?	1
HNF1A	12:120993533:AG:A	NM_000545.6:c.543del	p.Gln182Argfs*4	1
HNF1A	12:120993563:AG:A	NM_000545.8:c.572del	p.Gly191ValfsTer42	1
HNF1A	12:120993639:C:T	NM_000545.8:c.646C>T	p.Gln216Ter	1
HNF1A	12:120993678:C:T	NM_000545.6:c.685C>T	p.Arg229*	9
HNF1A	12:120993678:CGA:C	NM_000545.8:c.690_691del	p.Glu230AspfsTer8	1
HNF1A	12:120993701:C:CA	NM_000545.6:c.710dup	p.Asn237Lysfs*2	1
HNF1A	12:120994162:A:G	NM_000545.8:c.714-2A>G	p.?	1
HNF1A	12:120994297:AT:A	NM_000545.8:c.848del	p.Met283ArgfsTer59	1
HNF1A	12:120994300:G:GACACGTAC	NM_000545.6:c.852_859dup	p.Ser287Thrfs*58	1
HNF1A	12:120994308:C:G	NM_000545.6:c.858C>G	p.Tyr286*	2
HNF1A	12:120996260:A:C	NM_000545.6:c.956-2A>C	p.?	2
HNF1A	12:120996261:G:C	NM_000545.8:c.956-1G>C	p.?	1
HNF1A	12:120996332:GAC:G	NM_000545.8:c.1028_1029del	p.Thr343SerfsTer75	1
HNF1A	12:120996358:TG:T	NM_000545.6:c.1053del	p.Ser352Profs*12	3
HNF1A	12:120996415:T:C	NM_000545.6:c.1107+2T>C	p.?	1
HNF1A	12:120996557:G:GC	NM_000545.6:c.1129dup	p.Leu377Profs*42	1
HNF1A	12:120996568:CCT:C	NM_000545.6:c.1136_1137del	p.Pro379Argfs*39	4
HNF1A	12:120996569:CT:C	NM_000545.6:c.1137del	p.Val380Serfs*4	4
HNF1A	12:120996572:TCAGCACCCTGA:T	NM_000545.8:c.1146_1156del	p.Leu383AlafsTer32	1
HNF1A	12:120996601:GA:G	NM_000545.8:c.1169del	p.Glu390GlyfsTer23	1
HNF1A	12:120996636:GA:G	NM_000545.6:c.1205del	p.Asn402Thrfs*11	1
HNF1A	12:120996670:A:ACC	NM_000545.6:c.1238_1239dup	p.Ile414Profs*44	1
HNF1A	12:120996672:CA:C	NM_000545.6:c.1240del	p.Ile414Serfs*43	0
HNF1A	12:120996707:C:CGTAG	NM_000545.6:c.1276_1277insAGGT	p.Phe426*	1
HNF1A	12:120996743:G:A	NM_000545.6:c.1309+1G>A	p.?	0
HNF1A	12:120997488:C:T	NM_000545.8:c.1324C>T	p.Gln442Ter	1
HNF1A	12:120997491:GCA:G	NM_000545.6:c.1330_1331del	p.Gln444Glufs*104	1
HNF1A	12:120997494:CAG:C	NM_000545.8:c.1333_1334del	p.Ser445CysfsTer103	1
HNF1A	12:120997525:G:GC	NM_000545.6:c.1362dup	p.Ser455Glnfs*94	1
HNF1A	12:120997560:C:T	NM_000545.6:c.1396C>T	p.Gln466*	0
HNF1A	12:120997620:C:T	NM_000545.6:c.1456C>T	p.Gln486*	1
HNF1A	12:120997647:CAGCTGCAG:C	NM_000545.6:c.1487_1494del	p.Leu496Profs*50	0

HNF1A	12:120999267:GC:G	NM_000545.8:c.1504del	p.Leu502SerfsTer29	2
HNF1A	12:120999390:G:A	NM_000545.8:c.1623+1G>A	p.?	2
HNF1A	12:120999576:G:GCCAGCATCCAGCA	NM_000545.8:c.1720_1732dup	p.Leu578GlnfsTer75	1
HNF1A	12:120999585:C:T	NM_000545.6:c.1726C>T	p.Gln576*	0
HNF1A	12:120999596:GCCGGCCCA:G	NM_000545.6:c.1743_1750del	p.His582Glnfs*64	1
HNF1A	12:120999628:G:C	NM_000545.6:c.1768+1G>C	p.?	1
HNF1A	12:121001070:TC:T	NM_000545.6:c.1776del	p.Ser593Alafs*67	1
HNF1A	12:121001080:TG:T	NM_000545.8:c.1786del	p.Val596CysfsTer64	1
HNF1A	12:121001097:TC:T	NM_000545.6:c.1802del	p.Ser601*	2
HNF1A	12:121001115:C:T	NM_000545.6:c.1819C>T	p.Gln607*	1
HNF1A	12:121001115:CAG:C	NM_000545.6:c.1822_1823del	p.Ser608Profs*40	4
HNF1A	12:121001132:A:AT	NM_000545.8:c.1837dup	p.Ser613PhefsTer36	1
HNF1B	17:37687394:T:C	NM_000458.4:c.1654-2A>G	p.?	0
HNF1B	17:37731709:G:A	NM_000458.4:c.931C>T	p.Gln311*	1
HNF1B	17:37731748:TG:T	NM_000458.4:c.891del	p.Tyr297*	1
HNF1B	17:37739439:C:A	NM_000458.4:c.544+1G>T	p.?	1
HNF4A	20:44355806:TGGTCAGCGTGAACGCGCCCCTC:T	NM_175914.4:c.5_26del	p.Val2Glyfs*95	1
HNF4A	20:44355841:G:T	NM_175914.4:c.37G>T	p.Glu13*	1
HNF4A	20:44406117:C:CTG	NM_175914.4:c.114_115dup	p.Ala39Valfs*66	1
HNF4A	20:44406145:CG:C	NM_175914.4:c.140del	p.Gly47fs	1
HNF4A	20:44406225:TC:T	NM_175914.4:c.219del	p.Cys74Alafs*30	1
HNF4A	20:44413693:G:A	NM_175914.4:c.320-1G>A	p.?	1
HNF4A	20:44413726:C:T	NM_175914.4:c.352C>T	p.Arg118*	1
HNF4A	20:44413740:T:G	NM_175914.4:c.366T>G	p.Tyr122*	1
HNF4A	20:44413795:C:T	NM_175914.4:c.421C>T	p.Arg141*	3
HNF4A	20:44414626:CA:C	NM_175914.4:c.547del	p.Ile183Serfs*41	1
HNF4A	20:44414646:TCC:T	NM_175914.4:c.570_571del	p.Leu191Glyfs*35	1
HNF4A	20:44414652:T:TG	NM_175914.4:c.574dup	p.Asp192Glyfs*35	1
HNF4A	20:44418509:CTAGG:C	NM_175914.4:c.669_670+2del	p.Gly224Glnfs*2	1
HNF4A	20:44419813:C:T	NM_175914.4:c.763C>T	p.Gln255*	2
HNF4A	20:44424098:GA:G	NM_175914.4:c.908del	p.Asp303Alafs*27	1
HNF4A	20:44424103:CCAGTATGACT:C	NM_175914.4:c.914_923del	p.Gln305Argfs*22	1
HNF4A	20:44428337:TCCCCCAG:T	NM_175914.4:c.1068_1074del	p.Pro357fs	2
HNF4A	20:44428469:C:T	NM_175914.4:c.1198C>T	p.Arg400*	1
HNF4A	20:44429562:C:G	NM_175914.4:c.1256C>G	p.Ser419*	1
INS	11:2159953:G:A	NM_001185098.1:c.232C>T	p.Gln78*	3
INS	11:2160784:C:G	NM_001185098.1:c.187+1G>C	p.?	0
INS	11:2160874:G:C	NM_001185098.1:c.98C>G	p.Ser33*	0
INS	11:2160921:C:T	NM_001185098.1:c.51G>A	p.Trp17*	0
KCNJ11	11:17387185:A:AGGTG	NM_000525.3:c.903_906dup	p.Ser303Hisfs*7	0
KCNJ11	11:17387278:G:GA	NM_000525.3:c.813_814insT	p.Pro272Serfs*37	0
KCNJ11	11:17387478:AGG:A	NM_000525.3:c.612_613del	p.Leu205Profs*57	0
NEUROD1	2:181677831:G:A	NM_002500.4:c.1030C>T	p.Arg344*	0
NEUROD1	2:181677963:TAA:T	NM_002500.4:c.896_897del	p.Phe299Tyrfs*39	0

NEUROD1	2:181678072:ATCAG:A	NM_002500.4:c.785_788del	p.Thr262Ilefs*68	0
NEUROD1	2:181678107:CTGCGCTGTAGGCG:C	NM_002500.4:c.741_753del	p.His247Glnfs*11	0
NEUROD1	2:181678197:G:A	NM_002500.4:c.664C>T	p.Gln222*	1
NEUROD1	2:181678244:T:TG	NM_002500.4:c.616dup	p.His206Profs*38	7
NEUROD1	2:181678432:CAG:C	NM_002500.4:c.427_428del	p.Leu143Alafs*55	0
NEUROD1	2:181678757:TCG:T	NM_002500.4:c.102_103del	p.His34Glnfs*30	0
NEUROD1	2:181678795:C:T	NM_002500.4:c.66G>A	p.Trp22*	0
NEUROD1	2:181678818:G:A	NM_002500.4:c.43C>T	p.Gln15*	0
PDX1	13:27920172:C:T	NM_000209.4:c.34C>T	p.Gln12*	0
PDX1	13:27920188:C:CA	NM_000209.4:c.51dup	p.Cys18Metfs*207	1
PDX1	13:27920192:C:A	NM_000209.4:c.54C>A	p.Cys18*	1
PDX1	13:27920320:GC:G	NM_000209.4:c.188del	p.Pro63Argfs*60	1
PDX1	13:27920348:G:GC	NM_000209.4:c.217dup	p.Leu73Profs*152	1
PDX1	13:27920348:GC:G	NM_000209.4:c.217del	p.Leu73Serfs*50	0
PDX1	13:27920419:ACCCG:A	NM_000209.4:c.288_291del	p.Ala97Glyfs*25	0
PDX1	13:27920482:T:TC	NM_000209.4:c.346dup	p.Gln116Profs*109	0
PDX1	13:27920501:G:A	NM_000209.4:c.363G>A	p.Trp121*	0
PDX1	13:27920546:T:C	NM_000209.4:c.406+2T>C	p.?	0
PDX1	13:27924273:G:T	NM_000209.4:c.424G>T	p.Glu142*	0
PDX1	13:27924344:CCT:C	NM_000209.4:c.496_497del	p.Leu166Ilefs*58	0
PDX1	13:27924499:GT:G	NM_000209.4:c.651del	p.Gly218Alafs*12	1
PDX1	13:27924513:G:T	NM_000209.4:c.664G>T	p.Glu222*	0
PDX1	13:27924518:T:TGA	NM_000209.4:c.671_672dup	p.Gln225Serfs*6	0
PDX1	13:27924546:G:T	NM_000209.4:c.697G>T	p.Glu233*	1
PDX1	13:27924618:C:T	NM_000209.4:c.769C>T	p.Arg257*	0
PDX1	13:27924641:CCTTAGCGCGT:C	NM_000209.4:c.794_803del	p.Leu265Argfs*38	1
RFX6	6:116877348:C:T	NM_173560.4:c.73C>T	p.Gln25*	2
RFX6	6:116877434:CG:C	NM_173560.4:c.164del	p.Gly55Alafs*18	1
RFX6	6:116877919:A:AT	NM_173560.4:c.348dup	p.Lys117*	0
RFX6	6:116880544:G:A	NM_173560.4:c.381G>A	p.Trp127*	0
RFX6	6:116882384:TC:T	NM_173560.4:c.526del	p.Leu176Serfs*2	1
RFX6	6:116882406:C:CT	NM_173560.4:c.546dup	p.Gly183Trpfs*42	1
RFX6	6:116882419:GC:G	NM_173560.4:c.559del	p.His187Ilefs*27	0
RFX6	6:116882430:T:C	NM_173560.4:c.566+2T>C	p.?	0
RFX6	6:116916031:C:A	NM_173560.4:c.804C>A	p.Tyr268*	0
RFX6	6:116916208:A:AT	NM_173560.4:c.872dup	p.Leu291Phefs*10	0
RFX6	6:116916217:T:G	NM_173560.4:c.875T>G	p.Leu292*	2
RFX6	6:116916251:T:TG	NM_173560.4:c.909_910insG	p.Pro304Alafs*10	0
RFX6	6:116919142:T:G	NM_173560.4:c.1028T>G	p.Leu343*	2
RFX6	6:116919165:A:T	NM_173560.4:c.1051A>T	p.Lys351*	0
RFX6	6:116919215:TCTAA:T	NM_173560.4:c.1104_1107del	p.Asp370Argfs*13	1
RFX6	6:116919224:CAAGAA:C	NM_173560.4:c.1113_1117del	p.Lys371Asnfs*30	0
RFX6	6:116919243:C:T	NM_173560.4:c.1129C>T	p.Arg377*	0

RFX6	6:116919267:C:T	NM_173560.4:c.1153C>T	p.Arg385*	0
RFX6	6:116920455:G:A	NM_173560.4:c.1327+1G>A	p.?	0
RFX6	6:116922117:G:A	NM_173560.4:c.1403G>A	p.Trp468*	0
RFX6	6:116923154:TC:T	NM_173560.4:c.1486del	p.Leu496Cysfs*2	0
RFX6	6:116924686:C:T	NM_173560.4:c.1573C>T	p.Arg525*	1
RFX6	6:116925497:CT:C	NM_173560.4:c.1724del	p.Leu575Argfs*15	0
RFX6	6:116927095:C:T	NM_173560.4:c.1954C>T	p.Arg652*	0
RFX6	6:116927460:TAC:T	NM_173560.4:c.2321_2322del	p.Thr774Argfs*22	0
RFX6	6:116928853:G:GC	NM_173560.4:c.2499dup	p.Tyr834Leufs*3	0
RFX6	6:116931366:TC:T	NM_173560.4:c.2650del	p.Gln884Asnfs*57	1
RFX6	6:116931454:CTT:C	NM_173560.4:c.2737_2738del	p.Leu913Thrfs*25	0
RFX6	6:116931457:T:TA	NM_173560.4:c.2739dup	p.Pro914Thrfs*25	0

ESM Table 4: Power calculations

Genes	Cohort	MAF	Power	Minimum detectable odds ratio
ABCC8, *HNF1B*, *INS*, *NEUROD1*, *KCNJ11*, *PDX1*, *RFX6*	tNGS (n=2,571)	0.0001	0.8	9.8
GCK	Sanger+tNGS (n=3,512)	0.0001	0.8	8.3
HNF1A	Sanger+tNGS (n=3,863)	0.0001	0.8	7.9
HNF4A	Sanger+tNGS (n=3,073)	0.0001	0.8	8.9

ESM Table 5: Gene burden tests for *GCK*, *HNF1A*, *HNF4A* in the MODY cohort (n=5,171) with UK Biobank (n=155,501) as controls

Variant type	Gene	Allele count in MODY cohort	Allele count in population cohort (UK Biobank)	P value	Odds ratio	95% CI lower limit	95% CI upper limit
**NMD-triggering PTV (MAF < 0.0001)**	*GCK*	125	17	2.78×10^{-188}	341.34	204.69	604.84
	HNF1A	74	7	8.92×10^{-112}	441.18	203.69	1135.62
	HNF4A	23	2	1.13×10^{-37}	584.1	144.26	5110.68
**NMD-triggering Synonymous (MAF < 0.0001)**	*GCK*	8	236	0.3	1.55	0.66	3.1
	HNF1A	18	517	0.2	1.44	0.85	2.3
	HNF4A	9	358	0.4	1.27	0.58	2.44
**NMD-escape PTV (MAF < 0.0001)**	*GCK*	11	1	5.22×10^{-18}	502.13	72.95	21609.47
	HNF1A	12	5	5.43×10^{-18}	99.33	32.56	359.97
	HNF4A	2	1	0.001	101.24	5.27	5972.06
**Non-NMD region: Synonymous (MAF < 0.0001)**	*GCK*	2	34	0.2	2.68	0.31	10.46
	HNF1A	5	92	0.08	2.25	0.71	5.44
	HNF4A	1	61	1	0.83	0.02	4.79

2,571 individuals had tNGS. 2,600 individuals underwent Sanger sequencing for either *GCK* (n = 941), *HNF1A* (n = 1,292), or *HNF4A* (n = 602).

ESM Table 6: Gene burden test (MAF < 0.0001) in MODY cohort (n=2,571) with UK Biobank (n=155,501) as controls

Variant type	Gene	Allele count in MODY cohort	Allele count in population cohort (UK Biobank)	*P* value	Odds ratio	95% CI lower limit	95% CI upper limit
**NMD-triggering PTV**	*ABCC8*	6	131	0.03	2.77	0.99	6.19
	HNF1B	3	0	$4.31x10^{-6}$	Inf	24.98	Inf
	INS	0	2	1	0.00	0.00	322.22
	PDX1	1	1	0.03	62.94	0.8	4940.62
	RFX6	11	38	$3.55 x10^{-10}$	17.49	8.06	34.94
**NMD-escape PTV**	*ABCC8*	0	6	1	0	0	51.28
	HNF1B	0	1	1	0	0	2273.75
	INS	3	1	$1.70x10 x10^{-5}$	180.88	14.57	8809.85
	KCNJ11	0	3	1	0	0	146.11
	NEUROD1	8	23	$2.80x10^{-8}$	21.02	8.13	48.72
	PDX1	6	37	0.0001	10.22	3.52	24.42
	RFX6	1	5	0.09	12.09	0.26	108.27
**NMD-triggering synonymous**	*ABCC8*	20	1110	0.64	1.09	0.66	1.69
	HNF1B	5	378	0.84	0.8	0.26	1.88
	INS	0	47	1	0	0	4.94
	PDX1	2	93	0.67	1.3	0.16	4.83
	RFX6	6	440	0.85	0.82	0.3	1.81
**NMD-escape synonymous**	*ABCC8*	0	36	1	0	0	6.52
	HNF1B	0	0	1	0	0	Inf
	INS	0	69	0.63	0	0	3.32
	KCNJ11	3	309	0.5	0.59	0.12	1.73
	NEUROD1	3	181	1	1.00	0.20	2.98
	PDX1	1	102	1	0.59	0.01	3.38
	RFX6	1	22	0.31	2.75	0.07	16.99

ESM Table 7: Sensitivity analysis gene burden test in MODY Sanger Sequencing cohort (n=2,600) with UK Biobank (n=155,501) as controls

Variant type	Gene	Allele count in MODY cohort	Allele count in population cohort (UK Biobank)	*P* value	Odds ratio	95% CI lower limit	95% CI upper limit
**NMD-triggering PTV (All)**	*GCK*	102	17	6.56×10^{-206}	991.82	588.36	1771.38
	HNF1A	39	7	3.54×10^{-74}	670.78	296.23	1777.76
	HNF4A	9	2	1.08×10^{-20}	1161.48	239.90	11048.85
**NMD-triggering PTV (MAF < 0.0001)**	*GCK*	102	17	6.56×10^{-206}	991.82	588.36	1771.38
	HNF1A	39	7	3.54×10^{-74}	670.78	296.23	1777.76
	HNF4A	9	2	1.08×10^{-20}	1161.48	239.90	11048.85
**NMD-escape PTV (All)**	*GCK*	10	1	6.95×10^{-22}	1653.03	234.84	71724.87
	HNF1A	7	5	1.99×10^{-12}	168.55	46.01	673.80
	HNF4A	2	1	4.46×10^{-5}	516.63	26.86	30475.23
**NMD-escape PTV (MAF < 0.0001)**	*GCK*	10	1	6.95×10^{-22}	1653.03	234.84	71724.87
	HNF1A	7	5	1.99×10^{-12}	168.55	46.01	673.80
	HNF4A	2	1	4.46×10^{-5}	516.63	26.86	30475.23

2,600 individuals underwent Sanger sequencing for either *GCK* (n = 941), *HNF1A* (n = 1,292), or *HNF4A* (n = 602).

ESM Table 8: Sensitivity analysis gene burden test (MAF < 0.00005) in MODY cohort (n=2,571) with UK Biobank (n=155,501) as controls

Variant type	Gene	Allele count in MODY cohort	Allele count in population cohort (UK Biobank)	*P* value	Odds ratio	95% CI lower limit	95% CI upper limit
**NMD-triggering PTV**	*ABCC8*	4	102	0.1	2.37	0.63	6.26
	HNF1B	3	0	4.31×10^{-6}	Inf	24.98	Inf
	INS	0	2	1	0	0	322.22
	PDX1	1	1	0.03	62.94	0.8	4940.62
	RFX6	11	38	3.55×10^{-10}	17.49	8.06	34.94
**NMD-escape PTV**	*ABCC8*	0	6	1	0	0	51.28
	HNF1B	0	1	1	0	0	2273.75
	INS	3	1	1.70×10^{-5}	180.88	14.57	8809.85
	KCNJ11	0	3	1	0	0	146.11
	NEUROD1	8	23	2.80×10^{-8}	21.02	8.13	48.72
	PDX1	5	17	1.97×10^{-5}	18.53	5.34	52.33
	RFX6	1	5	0.09	12.09	0.26	108.27
**NMD-triggering synonymous**	*ABCC8*	19	923	0.36	1.24	0.75	1.96
	HNF1B	3	281	0.64	0.64	0.13	1.9
	INS	0	47	1	0	0	4.94
	PDX1	2	93	0.67	1.3	0.16	4.83
	RFX6	4	246	1	0.98	0.27	2.55
**NMD-escape synonymous**	*ABCC8*	0	19	1	0	0	12.95
	HNF1B	0	0	1	0	0	Inf
	INS	0	23	1	0	0	10.52
	KCNJ11	1	197	0.39	0.31	0.008	1.73
	NEUROD1	2	122	1	0.99	0.12	3.66
	PDX1	1	85	1	0.71	0.02	4.07
	RFX6	1	22	0.31	2.75	0.07	16.99

ESM Table 9: Sensitivity analysis gene burden test (MAF < 0.0002) in MODY cohort (n=2,571) with UK Biobank (n=155,501) as controls

Variant type	Gene	Allele count in MODY cohort	Allele count in population cohort (UK Biobank)	*P* value	Odds ratio	95% CI lower limit	95% CI upper limit
**NMD-triggering PTV**	*ABCC8*	6	131	0.03	2.77	0.99	6.19
	HNF1B	3	0	4.31×10^{-6}	Inf	24.98	Inf
	INS	0	2	1	0	0	322.22
	PDX1	1	1	0.03	62.94	0.8	4940.62
	RFX6	11	38	3.55×10^{-10}	17.49	8.06	34.94
**NMD-escape PTV**	*ABCC8*	0	6	1	0	0	51.28
	HNF1B	0	1	1	0	0	2273.75
	INS	3	1	1.70×10^{-5}	180.88	14.57	8809.85
	KCNJ11	0	3	1	0	0	146.11
	NEUROD1	8	23	2.80×10^{-8}	21.02	8.13	48.72
	PDX1	6	37	0.0001	10.22	3.52	24.42
	RFX6	1	5	0.09	12.09	0.26	108.27
**NMD-triggering synonymous**	*ABCC8*	21	1295	1	0.98	0.6	1.51
	HNF1B	5	481	0.37	0.63	0.2	1.48
	INS	0	47	1	0	0	4.94
	PDX1	2	93	0.67	1.3	0.16	4.83
	RFX6	10	656	1	0.92	0.44	1.71
**NMD-escape synonymous**	*ABCC8*	0	80	0.64	0	0	2.85
	HNF1B	0	0	1	0	0	Inf
	INS	0	69	0.63	0	0	3.32
	PDX1	1	147	0.74	0.41	0.01	2.33
	KCNJ11	5	368	0.84	0.82	0.26	1.94
	NEUROD1	4	218	0.79	1.11	0.30	2.88
	RFX6	1	22	0.31	2.75	0.07	16.99

ESM Table 10: Sensitivity analysis gene burden test for all PTVs (no MAF threshold) in MODY cohort (n=2,571) with UK Biobank (n=155,501) as controls

Variant type	Gene	Allele count in MODY cohort	Allele count in population cohort (UK Biobank)	*P* value	Odds ratio	95% CI lower limit	95% CI upper limit
NMD-triggering PTV	*ABCC8*	6	131	0.03	2.77	0.99	6.19
	HNF1B	3	0	4.31E-06	Inf	24.98	Inf
	INS	0	2	1	0.00	0.00	322.22
	PDX1	1	1	0.03	62.94	0.8	4940.62
	RFX6	11	38	3.55E-10	17.49	8.06	34.94
NMD-escape PTV	*ABCC8*	0	6	1	0.00	0.00	51.28
	HNF1B	0	1	1	0.00	0.00	2273.75
	INS	3	1	1.70E-05	180.88	14.57	8809.85
	KCNJ11	0	3	1	0	0	146.11
	NEUROD1	8	23	2.80×10^{-8}	21.02	8.13	48.72
	PDX1	6	37	0.0001	10.22	3.52	24.42
	RFX6	1	5	0.09	12.09	0.26	108.27

ESM table 11: Sensitivity analysis gene burden test (MAF < 0.0001) in MODY cohort (n=2,571) gnomAD v3.1.1 (n=34,029) as controls

Variant type	Gene	Allele count in MODY cohort	Allele count in population cohort (gnomAD)	*P* value	Odds ratio	95% CI lower limit	95% CI upper limit
NMD-triggering PTV	*ABCC8*	6	26	0.02	3.05	1.03	7.59
	GCK	126	5	3.62×10^{-109}	72.06	43.23	127.67
	HNF1A	74	3	2.37×10^{-70}	225.87	74.32	1120.23
	HNF1B	14	2	7.63×10^{-15}	92.62	21.26	822.94
	HNF4A	23	2	3.21×10^{-23}	127.82	31.57	1118.39
	INS	0	1	1	0	0	511.79
	PDX1	1	0	0.07	Inf	0	Inf
	RFX6	9	9	1.14×10^{-6}	13.23	4.65	37.60
NMD-escape PTV	*ABCC8*	0	0	.	.	.	.
	GCK	11	0	4.77×10^{-12}	Inf	27.79	Inf
	HNF1A	11	1	1.03×10^{-10}	99.62	14.47	4287.16
	HNF1B	0	0	.	.	.	.
	HNF4A	2	2	0.04	11.08	0.80	152.82
	INS	3	0	0.0003	Inf	5.47	Inf
	KCNJ11	0	2	1	0	0	70.39
	NEUROD1	8	6	1.22×10^{-6}	17.63967	5.36571	61.70326
	PDX1	5	5	2.21×10^{-7}	62.99	14.49	273.78
	RFX6	1	6	0.4	2.20	0.05	18.18
NMD-triggering synonymous	*ABCC8*	17	260	0.6	0.86	0.49	1.41
	GCK	7	70	1.00	0.97	0.38	2.1
	HNF1A	17	120	0.3	1.28	0.72	2.14
	HNF1B	4	68	0.82	0.78	0.21	2.08
	HNF4A	9	79	0.4	1.26	0.56	2.52
	INS	0	8	1.00	0.00	0.00	7.75
	PDX1	2	24	0.70	1.10	0.13	4.44
	RFX6	8	98	0.9	1.08	0.45	2.21
NMD-escape synonymous	*ABCC8*	0	10	1.00	0.00	0.00	5.90
	GCK	3	12	0.2	2.42	0.44	8.97
	HNF1A	3	12	0.2	2.26	0.41	8.38
	HNF1B	0	1	1.00	0.00	0.00	511.84
	HNF4A	1	13	1.00	0.85	0.02	5.67
	INS	0	12	1.00	0.00	0.00	4.76
	KCNJ11	2	70	0.24	0.38	0.04	1.42
	NEUROD1	3	59	0.80	0.67	0.13	2.06
	PDX1	1	25	1.00	0.53	0.01	3.23
	RFX6	1	4	0.3	3.31	0.07	33.41

2,571 individuals had tNGS. 2,600 individuals underwent Sanger sequencing for either *GCK* (n = 941), *HNF1A* (n = 1,292), or *HNF4A* (n = 602).

ESM Table 12: List of *INS* missense variants in patients used for comparison with patients with NMD-escape PTVs in *INS*

Variant	Number of individuals
p.R6C	1
p.A24D	1
p.A24V	1
p.H29Q	2
p.L30V	1
p.G32R	1
p.G32S	3
p.C43F	2
p.G44R	1
p.R46Q	9
p.R55C	9
p.S85C	2
p.R89C	5
p.C96S	1
p.C96Y	4

ESM Table 13: Case reports published about *INS* NMD-escape LoF variants in young-onset diabetes

Variant	Ref	Study type	No. of families	No. of cases with variant/No. of cases with diabetes	Age of diagnosis	Sex	Treatment	LOD score	Notes
p.Glu57*	PMID: 34362814	Cohort	1	1/1	Not available	Not available	Not available	n/a	-
p.Gly69Aalfs*62	PMID: 38553172	Case report	1	4/4	13,14,17, 22	3 F, 1 M	Metformin + Gliclazide + FGM, CSII MM780G, Insulin + FGM, Sitagliptin/Metformin + Insulin.	n/a	All individuals negative for all antibodies tested.
p.Gly71Alafs*60	PMID: 36504295	Cohort	1	1/1	7	M	Not available	n/a	Proband has no affected parents.
p.Gly73Trpfs*?	PMID: 30182532	Case report	1	5/7	15, 36, others not available	4 F, 3 M	Insulin + Metformin, Insulin, Gliclazide + Metformin, Dialysis + No Diabetes meds, Gliclazide + Metformin.	1.2	No diabetes in 2 carriers- one is 83 years old, other is 9.
p.Gln78Argfs*53	PMID: 25721872	Case report	1	3/3	14, 32, 26	1 F, 2 M	Insulin, Diet, Metformin + Insulin + Detemir + Sitagliptin + Glimepirid.	n/a	Proband- GAD, IA2 negative.
p.Cys96Valfs*35	PMID: 32086287	Cohort	1	1/1	Not available	Not available	Not available	n/a	-

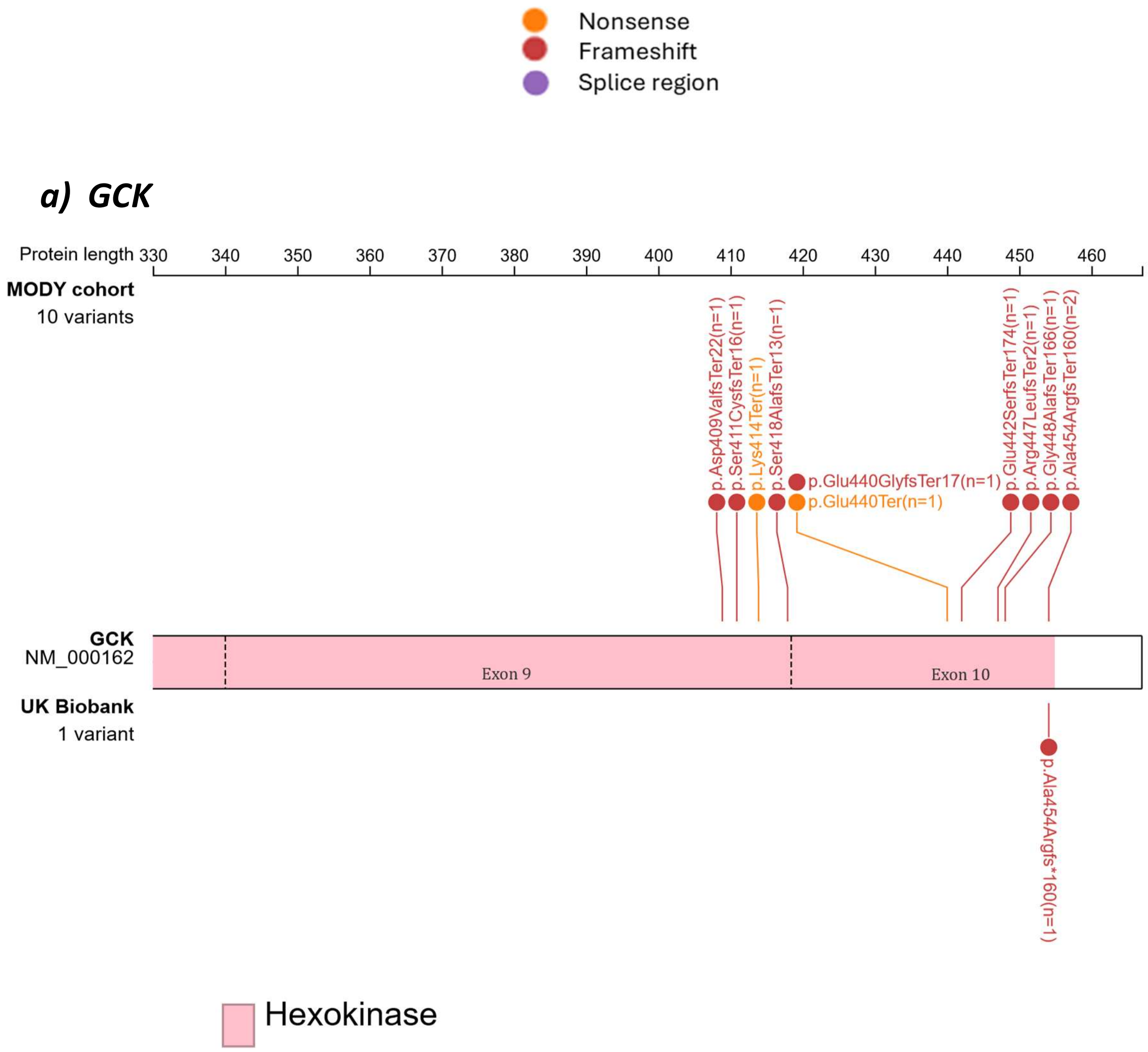

Nonsense
Frameshift
Splice region
a) GCK
Protein length
330
340
350
360
370
380
390
400
410
420
430
440
450
460
MODY cohort
10 variants
p.Asp409ValfsTer22(n=1)
p.Ser411CysfsTer16(n=1)
p.Lys414Ter(n=1)
p.Ser418AlafsTer13(n=1)
p.Glu440GlyfsTer17(n=1)
p.Glu440Ter(n=1)
p.Glu442SerfsTer174(n=1)
p.Arg447LeufsTer2(n=1)
p.Gly448AlafsTer166(n=1)
p.Ala454ArgfsTer160(n=2)
GCK
NM_000162
Exon 9
Exon 10
UK Biobank
1 variant
p.Ala454Argfs*160(n=1)
Hexokinase

b) HNF1A

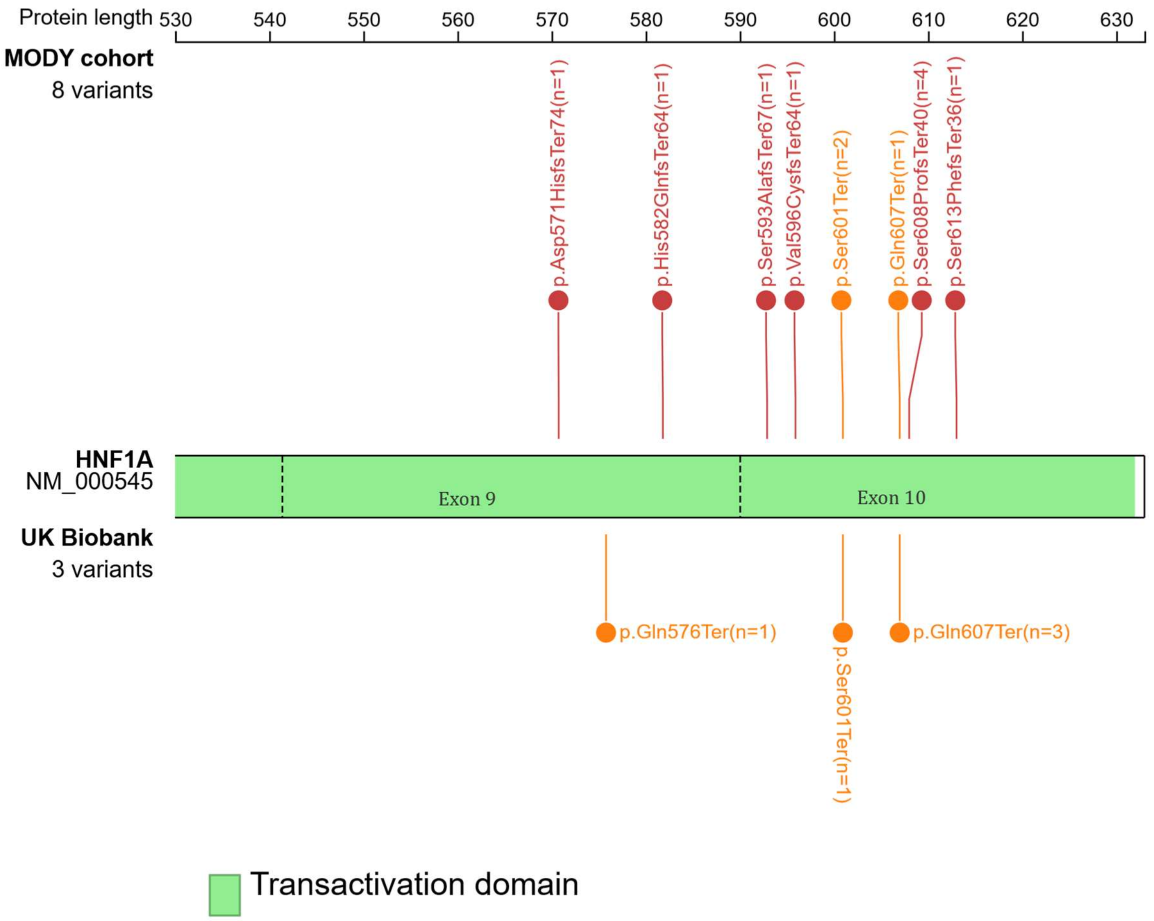

Protein length
530
540
550
560
570
580
590
600
610
620
630
MODY cohort
8 variants
p.Asp571HisfsTer74(n=1)
p.His582GlnfsTer64(n=1)
p.Ser593AlafsTer67(n=1)
p.Val596CysfsTer64(n=1)
p.Ser601Ter(n=2)
p.Gln607Ter(n=1)
p.Ser608ProfsTer40(n=4)
p.Ser613PhefsTer36(n=1)
HNF1A
NM_000545
Exon 9
Exon 10
UK Biobank
3 variants
p.Gln576Ter(n=1)
p.Ser601Ter(n=1)
p.Gln607Ter(n=3)
Transactivation domain

c) *HNF4A*

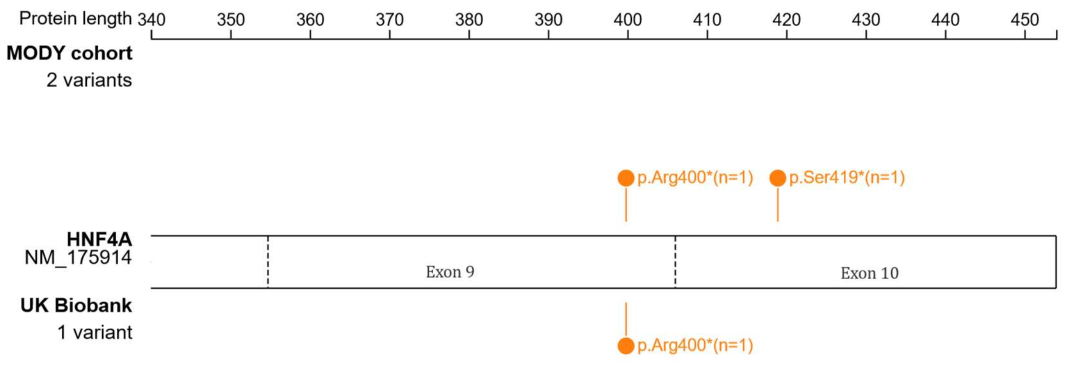

d) *PDX1*

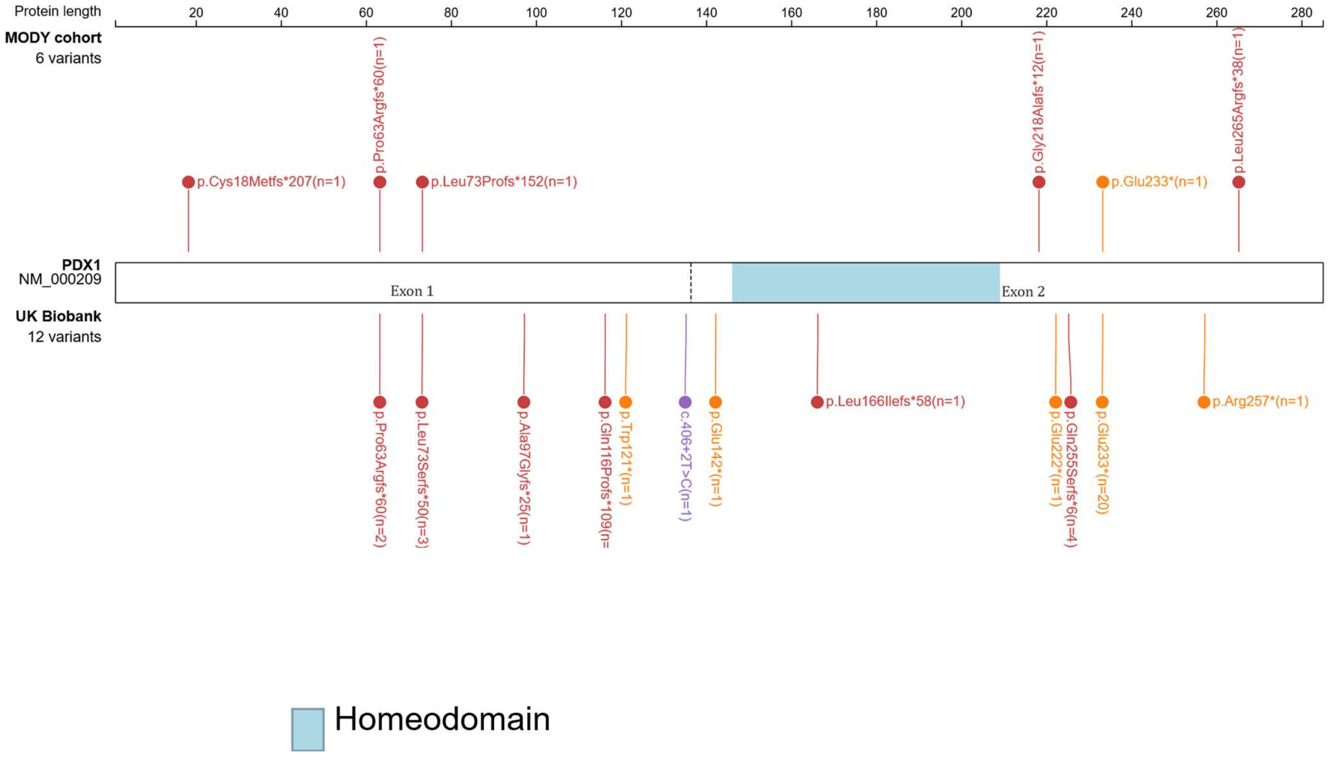

e) RFX6

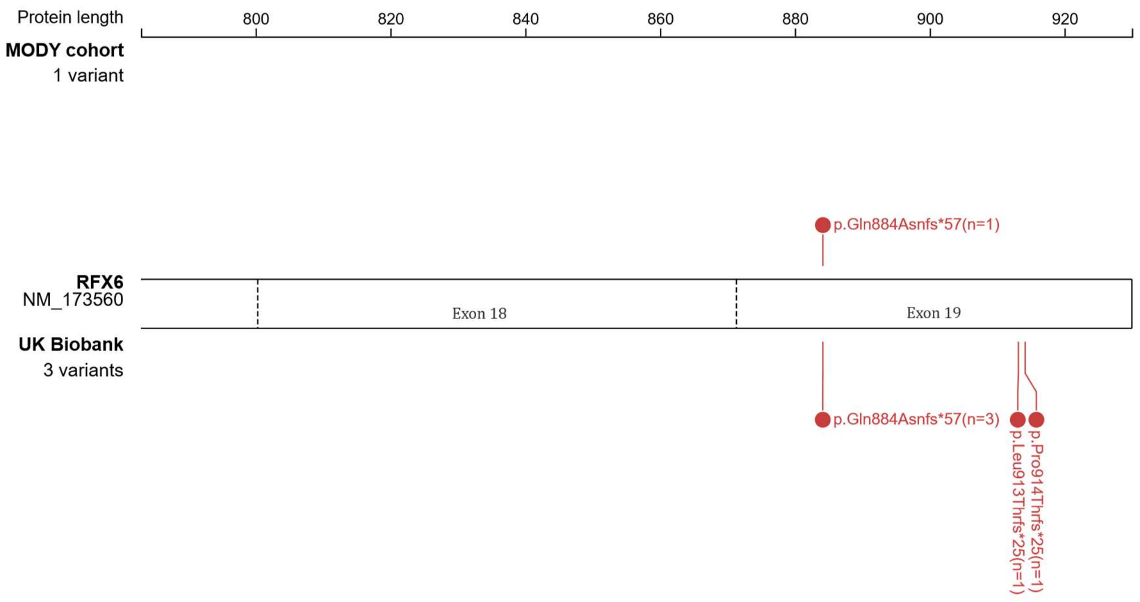

ESM Fig 1: Figure showing NMD-escape PTVs in MODY genes. This includes NMD-escape LOF variants split into nonsense, frameshift and splice region variants. The functional domains within each gene have been indicated. Variants identified in the MODY cohort are shown above the gene, while those from the UK Biobank are displayed below. Figures were generated using ProteinPaint (https://proteinpaint.stjude.org/).

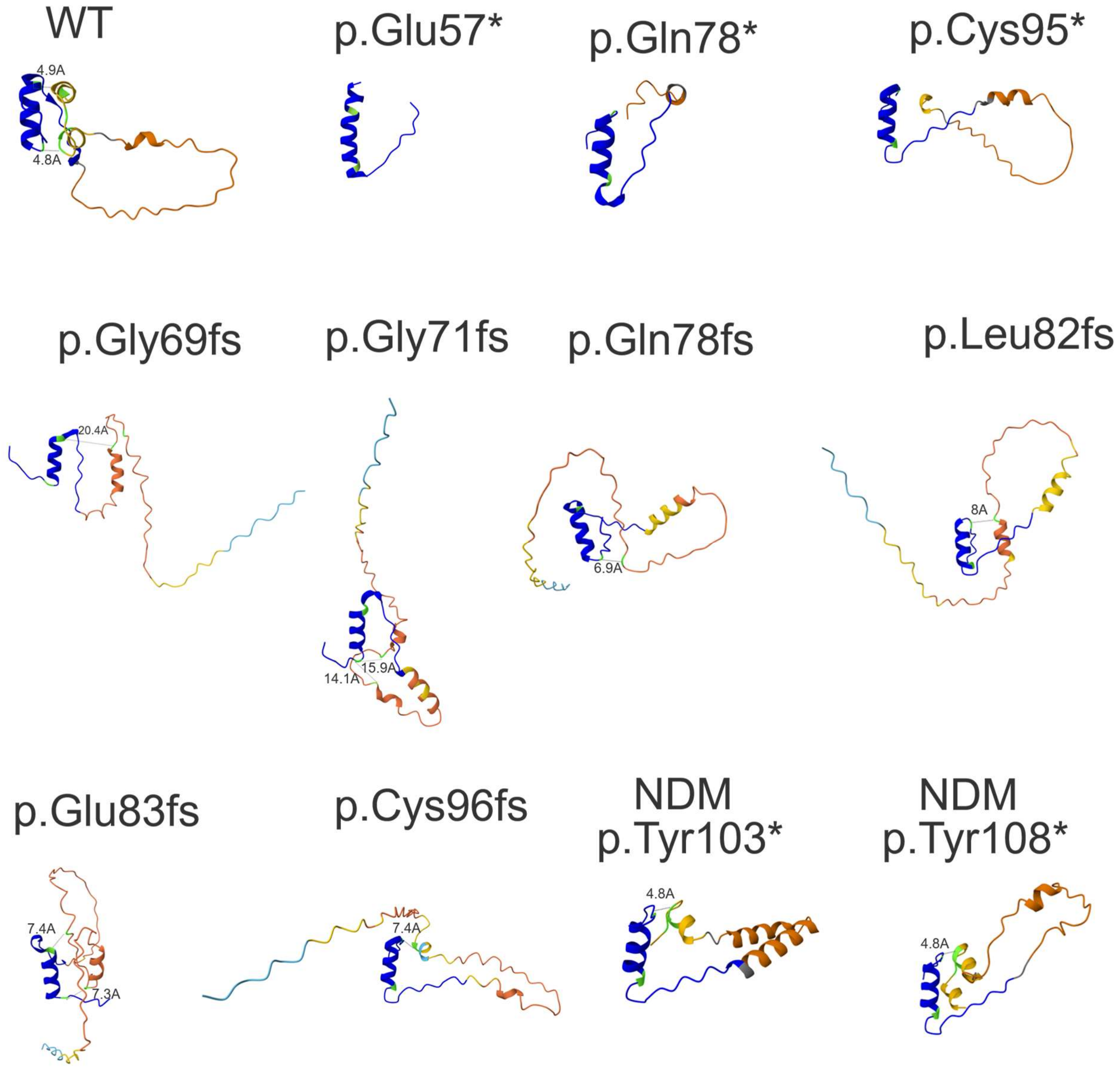

ESM Fig. 2: Protein predictions for *INS* NMD-escape. Proteins were modelled using Alphafold. B chains are shown in dark blue. Cysteines are shown in green and predicted distances between cysteines are measured in Angstrom and shown in the plots.

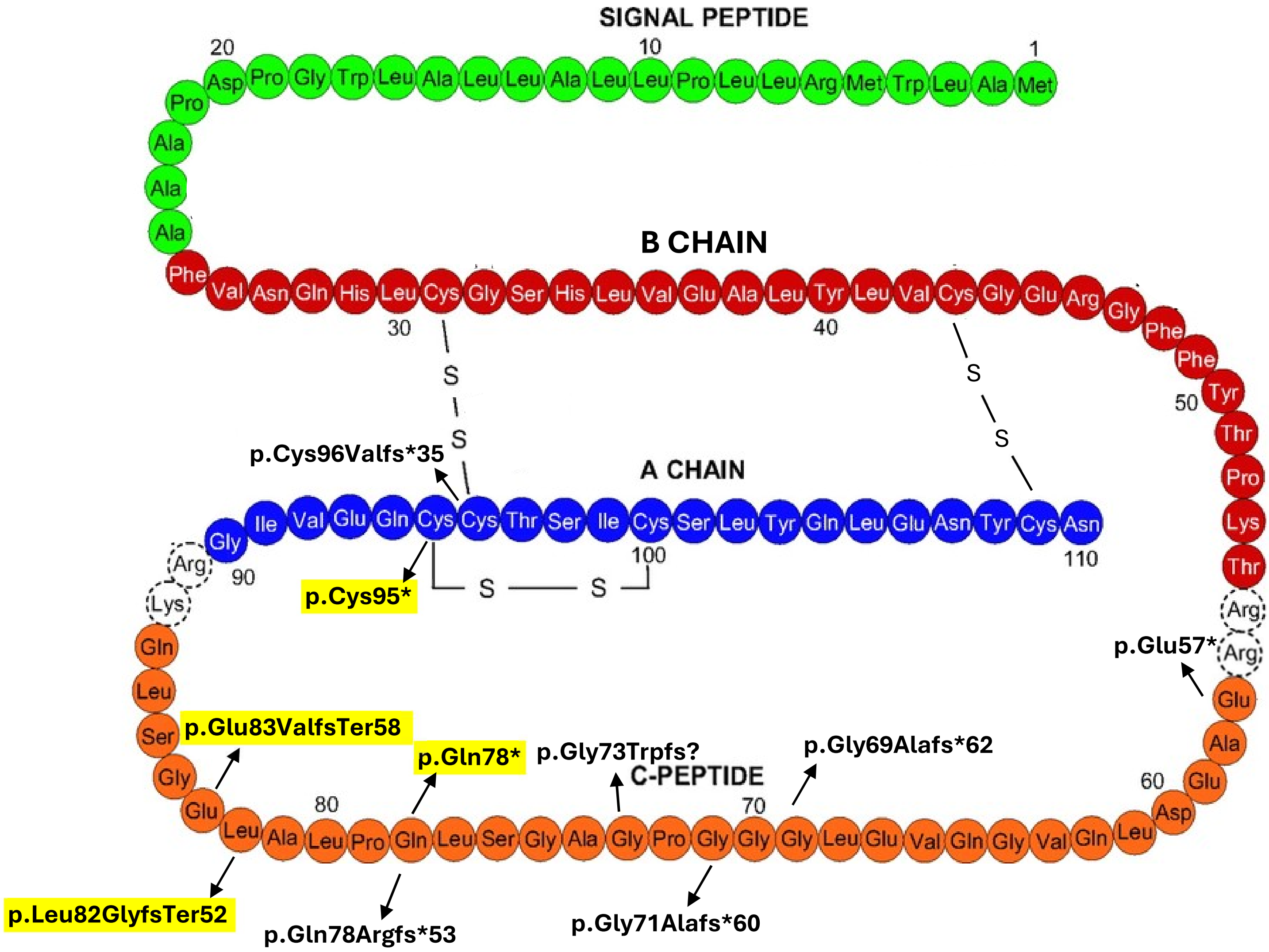

ESM Fig. 3: Figure showing NMD-escape PTVs in *INS*. The structure of preproinsulin is shown in the figure, with signal peptide in green, B chain in red, C peptide in orange and A chain in blue, with 3 disulphide bonds shown. Highlighted variants are seen in the MODY cohort, others reported in literature. (Figure adapted from https://doi.org/10.1016/j.molmet.2021.101280)
